# *Wearanize+*: a multimodal dataset for evaluating wearable technologies in sleep research

**DOI:** 10.1093/sleepadvances/zpaf094

**Published:** 2025-12-27

**Authors:** Niloy Sikder, Lieuwe Verkaar, Anastasiya Paltarzhytskaya, Selin Acan, Leonore Bovy, Tatiana Almazova, Elena Krugliakova, Yevgenia Rosenblum, Matthias Krauledat, Martin Dresler, Paul Zerr

**Affiliations:** Radboud University Medical Center, Donders Institute for Brain, Cognition and Behaviour, Nijmegen, The Netherlands; Faculty of Technology and Bionics, Rhine-Waal University of Applied Sciences, Kleve, Germany; Radboud University Medical Center, Donders Institute for Brain, Cognition and Behaviour, Nijmegen, The Netherlands; Radboud University Medical Center, Donders Institute for Brain, Cognition and Behaviour, Nijmegen, The Netherlands; Radboud University Medical Center, Donders Institute for Brain, Cognition and Behaviour, Nijmegen, The Netherlands; Radboud University Medical Center, Donders Institute for Brain, Cognition and Behaviour, Nijmegen, The Netherlands; Radboud University Medical Center, Donders Institute for Brain, Cognition and Behaviour, Nijmegen, The Netherlands; Radboud University Medical Center, Donders Institute for Brain, Cognition and Behaviour, Nijmegen, The Netherlands; Radboud University Medical Center, Donders Institute for Brain, Cognition and Behaviour, Nijmegen, The Netherlands; Faculty of Technology and Bionics, Rhine-Waal University of Applied Sciences, Kleve, Germany; Radboud University Medical Center, Donders Institute for Brain, Cognition and Behaviour, Nijmegen, The Netherlands; Radboud University Medical Center, Donders Institute for Brain, Cognition and Behaviour, Nijmegen, The Netherlands

**Keywords:** sleep dataset, wearable data, sleep EEG, polysomnography, multimodal dataset

## Abstract

Sleep research heavily relies on polysomnography recordings to assess sleep architecture. While effective, this method is time-consuming and requires substantial resources and labor. Modern wearable devices provide a promising alternative for sleep monitoring as they are easy to wear and maintain. However, these devices are constrained by a limited number of channels and comparatively lower data quality, which often leads to unreliable outcomes derived from partial readings. To address this, we propose using multiple wearable devices and combining their outputs to acquire reliable sleep data. However, the feasibility of this approach must be rigorously tested before being relied upon to supplant polysomnography in scientific studies. To facilitate this, we have curated a dataset with concurrent full polysomnography and wearable device recordings of overnight sleep sessions. This dataset, named *Wearanize+*, comprises data from 130 healthy (mostly young adult) participants, one night each, collected at home using three wearable devices: *Zmax* headband, *Empatica E4* wristband, and *ActivPAL* leg patch, alongside polysomnography. It also includes questionnaires providing information on participants’ sleep, dreams, and overall health. This paper documents the setup, data collection, and preprocessing steps of the Wearanize+ project, serving as a guide to using the resulting dataset. Our objectives with the dataset include developing machine learning models that can derive polysomnography-grade sleep stages from wearable devices’ data and exploring alternative data modalities for sleep-stage scoring, particularly when EEG signals are excessively noisy. The Wearanize+ dataset is available via the *Radboud Data Repository*.

*This paper is part of the Consumer Sleep Technology Collection*.

**Statement of Significance**

The Wearanize+ dataset is a multimodal dataset containing overnight sleep recordings from 130 healthy adults, combining concurrent polysomnography with three wearables (Zmax headband, Empatica E4 wristband, and ActivPAL leg patch) and sleep and health questionnaires. The synchronized dataset is suitable for device-specific validation, development of machine learning models to infer polysomnography (PSG)-grade sleep stages from wearable data, and systematic evaluation of alternative modalities and multidevice fusion. By supporting rigorous validation and scalable model development, the dataset can help bridge the gap between wearable data and gold-standard PSG for large-scale, clinical sleep research.

## Introduction

Polysomnography (PSG) is a comprehensive sleep assessment protocol used to study sleep in healthy individuals and to diagnose sleep disorders. PSG simultaneously records several physiological signals during sleep, including electroencephalography (EEG), electrooculography (EOG), electromyography (EMG), and electrocardiography (ECG), and is considered the gold standard in sleep research [1, 2]. Such comprehensive monitoring enables researchers to examine various aspects of sleep, including sleep stages, slow waves, spindles, and anomalies [3, 4]. PSG devices differ in size, portability, and configuration, most notably in the number of channels. In most sleep research, 12–16-channel PSG systems are used, as recommended by the American Academy of Sleep Medicine (AASM) sleep-scoring manual [4]. The primary advantage of PSG is its comprehensiveness and standardization, allowing for precise diagnosis and understanding of complex sleep architecture. However, recording sleep with PSG has several drawbacks: the setup is time-consuming and cumbersome, requires trained personnel, and is expensive [5]. Moreover, many participants find sleeping in a lab while wearing a PSG system uncomfortable, which may alter their natural sleep behavior [6].

Wearable technology has made significant progress in recent years [7]. Wearable devices (*wearables*) are typically worn on various parts of the body and collect data through multiple sensors from those body parts. Many smartwatches, wristbands, smart rings, and chest monitors can record both sleep cycles and waking activities if worn throughout the day (and night). These devices primarily rely on photoplethysmography (PPG) to measure heart rate (HR) and other cardiovascular activity and on actigraphy (ACT) to discern rest–wake or sleep cycles. Many of them report various sleep metrics, including sleep quality and duration, number of awakenings at night, and sleep stages, using proprietary, black-box algorithms with varying degrees of accuracy. Since these sleep stages are PPG- and ACT-based, they often differ significantly from those derived from gold-standard PSG (EEG, EMG, EOG, and ECG), as evidenced by early studies with wearables [8–10]. However, they are increasingly being used in sleep studies, and some are showing promising results, indicating their usefulness [11–14]. Moreover, the World Sleep Society has recently issued best-practice recommendations on the use of wearables for sleep monitoring, underscoring their growing utility in research [15].

The primary advantage of wearables is their convenience—compared with PSG, they are inexpensive, unobtrusive, easy to use, and require minimal maintenance, enabling longitudinal and large-scale data collection in natural sleeping environments. Thus, wearables show the potential to address several drawbacks of PSG in sleep monitoring. Sleep headbands, capable of recording EEG, PPG, and ACT, are showing the most promise in this regard. Since headbands rely on EEG signals for analysis, they are closer to PSG than other wearables. Examples of sleep headbands include *Muse S* [16], *Zmax* [17], *SmartSleep* [18], *SleepLoop* [19], *OpenBCI* headband [20], *Bitbrain Ikon* [21], *Sleep Profiler* [22], *Dreem2* [23], *Frenz* [24], *Elemind* [25], *Somnee* [26], and *iBand+* [27]. Nevertheless, headbands usually lack the spatial resolution and accuracy provided by PSG, as they typically have two to four EEG channels, often interfaced to the forehead area via dry electrodes (exceptions include Zmax and SleepLoop), resulting in partial measurements with subpar data quality. Therefore, even after incorporating EEGs in wearables, their validity in general and clinical sleep research remains a topic of further study.

From a research perspective, many questions remain about the use of wearables in scientific studies. For example, if wearable headbands cannot match the quality of PSG, how accurate are they based on their partial view of brain activity? In addition to EEG channels, wearable headbands often provide PPG, ACT, and other information that basic PSG systems may lack. Can these additional modalities be considered alternative descriptors of sleep and used accordingly in sleep research? Does adding other modalities, such as electrodermal activity (EDA), HR variability (HRV), and skin temperature, enhance their utility? Combining the outputs of multiple wearables may provide a broader view. Can a specific set of devices, strategically placed on various parts of the body, be used to test specific hypotheses in lieu of a full PSG setup? EEG tends to be noisy, especially due to body movements. When EEG fails (or produces suboptimal data), can other modalities serve as substitutes? And lastly, can we map the outputs of wearables to those acquired from PSGs so that reliable sleep stages can be achieved when PSGs are unavailable, too difficult to apply, or too costly for a project?

We have created a multimodal dataset, named *Wearanize+*, to address these questions and more. Using this dataset, we aim to evaluate the potential of wearable technology for sleep analysis and to develop automated sleep-scoring models that achieve PSG-equivalent sleep stages. A project that will directly benefit from this dataset is the Healthy Brain Study (HBS), a longitudinal study (*N ≈ 850*) conducted by multiple research centers and based at the Radboud University campus in Nijmegen, the Netherlands [28]. It contains 3 weeks of sleep recordings from participants using Zmax (an EEG headband), *Empatica E4* (a wristband) [29], and *ActivPAL* (a leg patch) [30]. However, it lacks accompanying PSG, making it challenging to establish reliable sleep metrics, which has motivated the creation of this dataset.

## Materials and Methods

In this section, we describe the data collection procedure, devices used, and steps taken to preprocess the data. Before addressing these topics, we provide additional background information to contextualize the Wearanize+ dataset.

Collecting (simultaneous) data from PSG and a single wearable is quite common [31, 32]. Such studies usually fall into one of three categories: testing the device’s efficacy in diagnosing a specific disease, comparing the device’s outputs with those of PSG (also known as *validation studies*), or developing device-specific automatic sleep-stage scoring algorithms (also known as *autoscorers*). Examples of (recent) studies from the first category include the use of a wearable vest [33], a portable EEG device [34], and an HRV and SpO_2_-based wearable sleep monitor [35] for obstructive sleep apnea (OSA) detection. Studies utilizing smartwatches and wristbands, such as PPG readings from Empatica E4 [36] and Huawei Watch GT 2 [37, 38], have diagnosed OSA and respiratory events with varying degrees of success. The second category includes validation studies of the Zmax headband [39], Muse headband [40], *Fitbit Charge 3* wristband [41], *Fitbit Alta HR* wristband [42], *Respironics Actiwatch 2* [42], *Fatigue Science Readiband* [43], *Garmin Fenix 5S* and *Vivosmart 3* [43], *WHOOP* wristband [44], and *Basis B1 smartwatch* [45]. Studies often combine these objectives, offering a comprehensive examination of device reliability for OSA detection and their capability in sleep measurement [33, 34]. Lastly, some studies focus on developing autoscorers using the device’s data and PSG-based sleep scores as labels [46] or applying transfer learning methods to map the data to PSG-based scores [47].

Data collection using multiple wearables placed at different areas of the body with simultaneous PSG is less common, which is where this study differs from the previous ones. For instance, a 2020 study collected PSG and data from four wearables in a sleep lab [48]. The researchers conducted device-wise sleep–wake classification and compared various sleep metrics derived from them with their PSG-based counterparts. However, the sample size (*N = 8*) is considerably lower than that of most contemporary studies. Moreover, although the binary classifiers achieved high sensitivity, most showed low specificity. A 2023 study (*N = 75*) recorded PSG alongside 11 sleep trackers, totaling 3890 hours of sleep data (543 hours with parallel PSG) [49]. However, device usage varied among participants. However, the analysis showed varying degrees of agreement between PSG-based sleep scores and those provided by the devices based on their native algorithms (F1-scores ranging from 25.88% to 68.63%). As these examples underscore, there remains a need for large-scale, consistent studies that simultaneously monitor sleep using PSG and wearables to improve the reliability of wearables in advanced sleep research. We aim to address this gap with the presented dataset and subsequent research using it.

### Devices

The Wearanize+ dataset contains simultaneous recordings from 130 participants who wore a PSG system (*SOMNOscreen plus* [50] or *Mentalab Explore Pro* [51]) and three wearables: a Zmax headband, an Empatica E4 wristband, and an ActivPAL leg patch during a full night of sleep. Some participants also wore two additional experimental wearables: a smartwatch and a headband with functional near-infrared spectroscopy (fNIRS)-recording capabilities. As these devices are still in development, their readouts are not included in this dataset. The first three devices will be included to develop a method for reliably obtaining sleep scores in the absence of PSG for HBS sleep data. The dataset also includes responses to three widely used questionnaires: the *Pittsburgh Sleep Quality Index (PSQI)* [52], the *Mannheim Dream Questionnaire (MADRE)* [53], and the *Patient Health Questionnaire (PHQ-9)* [54]. For simplicity, in the remainder of the article, we will refer to SOMNOscreen plus, Mentalab Explore Pro, Empatica E4, and ActivPAL as *Somnoscreen*, *Mentalab*, *Empatica*, and *Activpal*, respectively.

#### PSG systems

Somnoscreen is a portable PSG system widely used in sleep research and has been used to validate the performance of many wearable devices [39, 55–58]. In addition to the fundamental modalities of PSG, Somnoscreen also records movement and body positions throughout the night. While most channels have a sampling rate of 256 Hz, movement and body position are recorded at 128 and 4 Hz, respectively. However, the Somnoscreen does not store the raw tri-axial accelerometer readings. A key challenge of using Somnoscreen is dealing with its limited battery life. Somnoscreen uses a 10.36 Wh Lithium-ion battery (Part No.: 110686-O [59]) that supports a recording time of 8–10 hours, which means the devices can run out of battery during longer sessions (and several participants have reported this). The initialization of the Somnoscreen recordings and the export of data require the use of the *Domino* software [60], which was also used to convert the raw DAT-format data to the more widely used European Data Format (EDF).

Mentalab is another portable EEG system widely utilized in sleep research and other neurophysiological studies. It offers configurations with up to 32 channels, which were used to collect data in this project at a sampling rate of 250 Hz. In addition to EEG, Mentalab is equipped with a 9-axis motion sensor (tri-axial gyroscope, accelerometer, and magnetometer) at a sampling rate of 20 Hz. We exported the raw data as CSV files and performed manual sleep scoring for our planned analysis. We used Mentalab to collect PSG data from 15 participants during the final phase of data collection to assess its usefulness in future studies.

#### Zmax EEG headband

EEG headbands have become more present in sleep research in the last decade [61, 62]. The Lite version [63] of Zmax used in this study has two gel-electrode-based frontal EEG channels, a tri-axial accelerometer, and PPG at a sampling rate of 256 Hz. The Zmax headband is compact, self-applicable, and suitable for regular overnight use with minimum maintenance. A recent study has validated its performance against the gold-standard PSG and, based on 900+ hours of simultaneous sleep recordings from 135 healthy participants, has found that the headband provides acceptable signal quality and does not significantly affect the participants’ sleep quality and mood, making it suitable for long-term sleep tracking [39]. However, similar to Somnoscreen, we have experienced issues with the Zmax devices’ battery life. They each replaced once during the project to increase recording time and mitigate other problems, such as broken hinges, detached straps, and the device being unresponsive or behaving unexpectedly.

#### Empatica wristband

Since its release in 2015, Empatica has become one of the most popular wrist-worn wearables. Due to its portability and ability to continuously collect high-quality biometric data, it has been extensively used in numerous research studies, including stress detection [64] and monitoring [65], estimation of HRV [66, 67], and measurement of EDA [68] and cardiac activity [69]. It records EDA, PPG (stored as blood volume pulse or BVP), tri-axial ACT, and skin temperature at 4, 64, 32, and 4 Hz, respectively. However, some additional measurements derived from BVP data, such as HR and the time between consecutive heartbeats (IBI), are also provided by the device.

#### Activpal leg patch

The Activpal leg patch from PAL Technologies [30] is a small, lightweight activity monitor designed to be worn on the thigh. This device is widely used to assess physical activity, sedentary behavior, and postural changes in free-living or clinical environments. It is usually worn directly on the skin using an adhesive patch or tape for a prolonged period of monitoring (up to a week) [70]. Activpal primarily records tri-axial ACT data at 20 Hz.

### Data collection procedure

The data collection methodology of this project followed a simple observational design without any interventions aimed at preserving participants’ natural sleep and recording them as comprehensively as possible. Our goal was to obtain one night of usable data (PSG and at least the first three devices) from each participant. The project was advertised through multiple channels, including leaflets and websites. The *SONA System* (Radboud Research Participation System) [71] was used for (formal) participant intake. Data collection was conducted in accordance with the Declaration of Helsinki [72], and the project was covered by the Donders Centre for Cognitive Neuroimaging blanket approval. All participants provided informed consent for their data to be used for research purposes and received a one-time remuneration of €50 for participation. Participants were only included if they met the following criteria:

1. Age: 18–50 years
2. Body mass index (BMI): ≥17.5
3. Not (primarily) a stomach sleeper
4. Not claustrophobic
5. No known history of sleep disorder or mental illness
6. Not currently taking any medications that affect sleep
7. No history of head or brain surgery
8. Not diagnosed with epilepsy
9. Not pregnant

**Figure 1:**
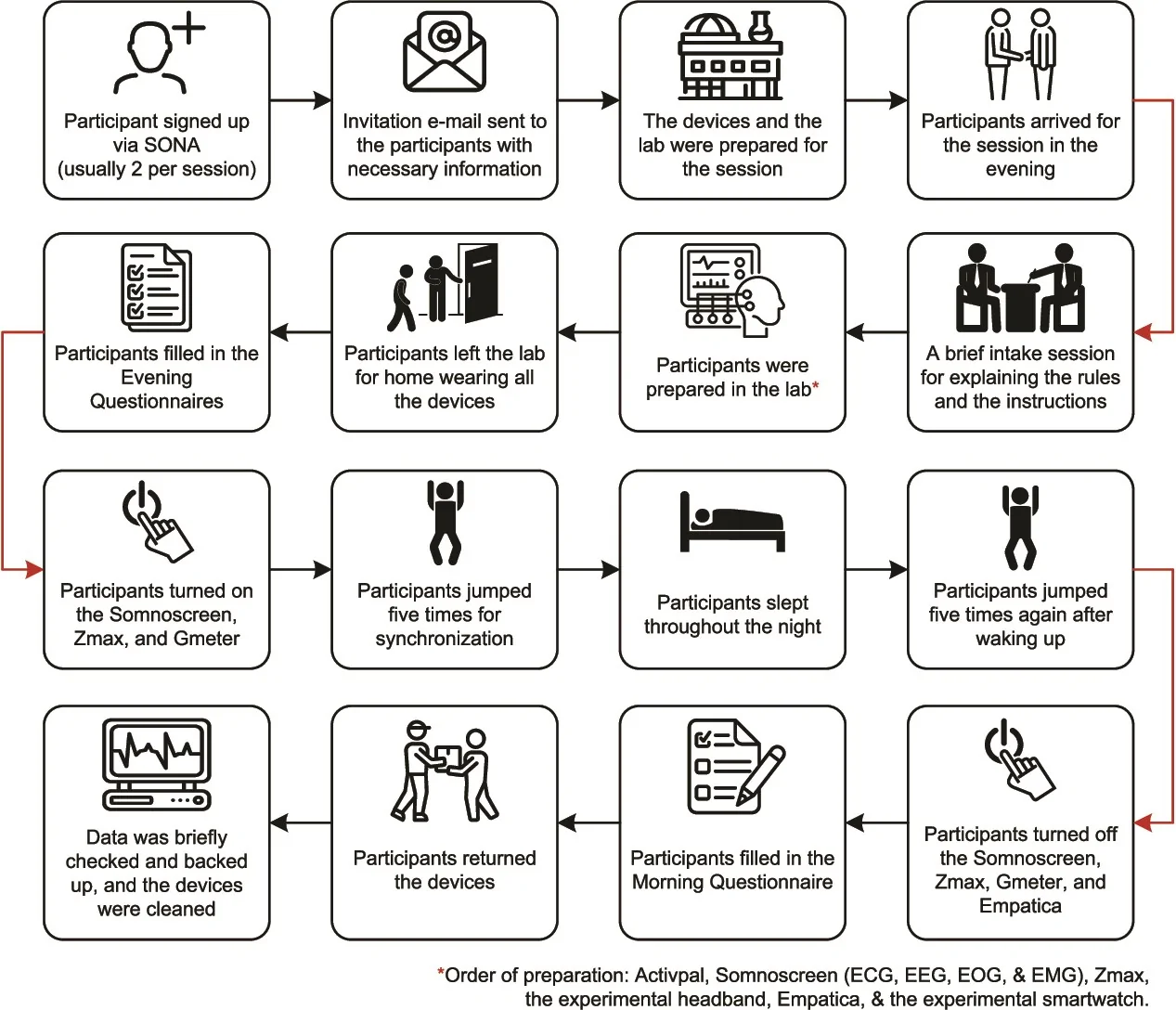
Workflow of the data collection procedure [74–87].

These measures ensured that the data collected would reflect the typical sleep behavior of healthy humans while adhering to ethical guidelines and scientific standards. The inclusion criteria and preparation guidelines (such as having an early dinner before the session and going to bed shortly after) were outlined on the study page in SONA [73] and communicated to the participants via an invitation email. Since the primary aim of the study was to generate a dataset for validating wearable systems under real-life conditions, we did not explicitly exclude participants who nap or have a history of night-shift work. We also did not restrict daytime consumption of caffeine or alcohol, but we prohibited both in the evening. The participants were instructed to avoid activities involving significant movement or sweating after the session to reduce the chances of electrodes becoming loose or disconnected. The workflow of preparing a participant and collecting data is outlined in Figure 1.

**Figure 2:**
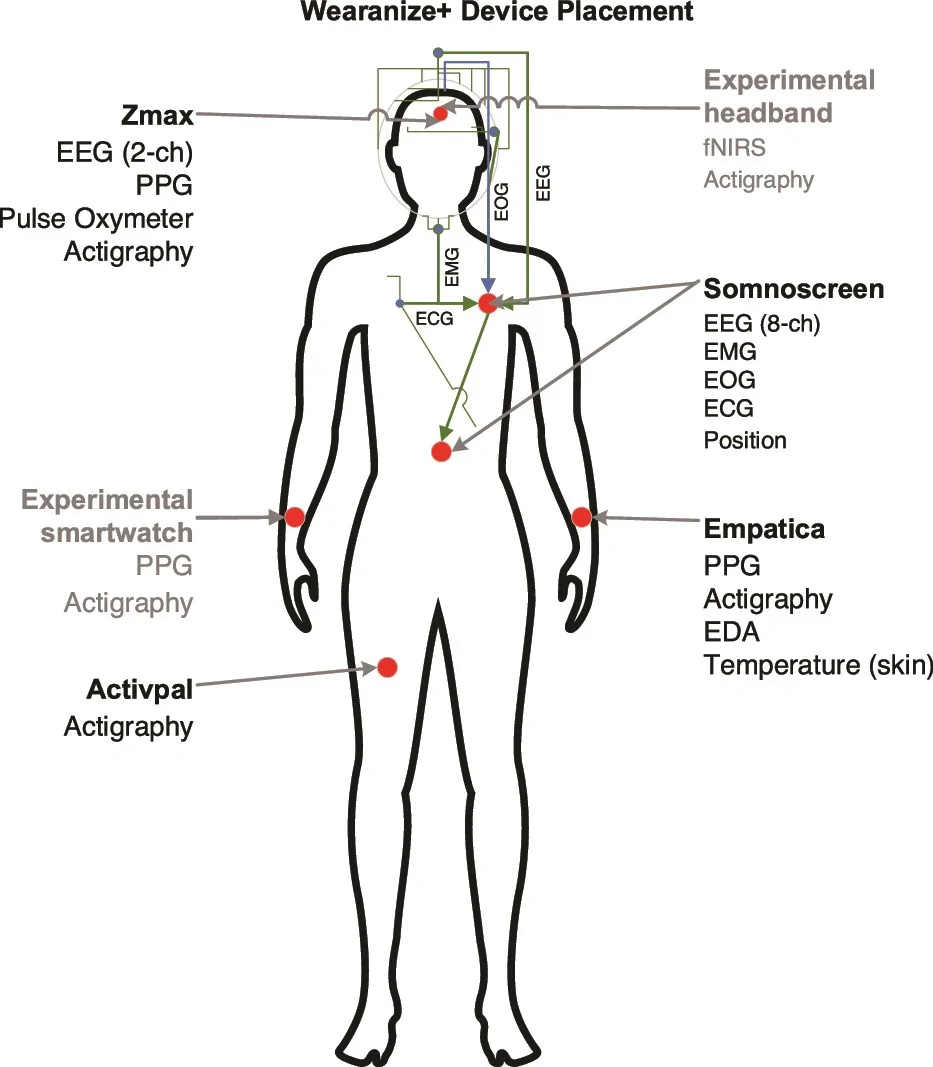
The positions of the devices on the participant’s body and their recording modalities [93]. Data from the devices shown in gray are not included in the current release.

Data collection sessions took place in the evening, typically from 18:00 to 20:00. Upon arrival, participants went through a short intake session where experimenters outlined the study’s goals, regulations, and participants’ responsibilities. Participants then signed an Informed Consent form and provided demographic information (such as gender, dominant hand, and birth year), which was recorded in the *Castor* platform [88]. This platform was also used to send the questionnaires to the participants and collect (and store) their responses. After the intake, participants proceeded to an EEG lab, where the experimenters prepared them for the overnight recording. Prior to the sessions, the experimenters prepared the lab with the necessary equipment required for the PSG setup, including *NuPrep* skin gel [89] (to clean dead cells off the contact points of the scalps), *Ten20* conductive paste [90] (to increase conductivity), *EC2+* electrode cream [91] (to fixate the scalp electrodes), and rubbing alcohol [92] (to clean the nonscalp contact points). Two complete sets of devices were charged and prepared for recording beforehand to minimize delay during the session. Figure 2 illustrates the placement of the devices on a participant’s body.

The Activpal leg patch was the first device placed on the participants. After initialization, the device was handed to them, and they were instructed to attach it to the front of their right thigh, one-third of the distance from the hip to the knee [94, 95], with some surgical tape [96] (by themselves in a separate room). They also put on an external accelerometer associated with the experimental headband in the front of their chest. The Somnoscreen devices we used have 10 EEG, three EMG, two EOG, and two ECG channels. Details about the types and placement positions of these electrodes are provided in Table 1. Before affixing the electrodes, the main module and the Digital Headbox of the Somnoscreen (which serves as the interfacing module between the electrodes and the main module) were positioned at the center of the lower abdomen and below the left shoulder on the chest, respectively, and were secured with their associated belts [101]. After that, the participant’s head size was measured, and the center point of the head was identified, which served as the reference point of the associated EEG cap. The cap was temporarily placed on the head to precisely mark the contact points of the scalp EEG electrodes with a marker, which was later removed.

**Table 1:** Placement positions of the Somnoscreen components for recording PSG

Component	Number	Electrode type	Placement position
EEG electrodes	10	Gold cup electrodes [97]	F3, F4, C3, C4, O1, O2, M1, M2, Ground (Gnd), and Ref, positioned according to the 10–20 International system of EEG electrode placement [98]
EMG electrodes	3	Gold cup electrodes	Two below the chin on the left and right sides, and the reference on the chin
EOG electrodes	2	Gold cup electrodes	One slightly above the right eye and the other slightly below the left eye
ECG electrodes	2	Patch electrodes [99, 100]	One 2 cm below the right collarbone and the other 2 cm below the left ribcage
Main module	1	—	The center of the lower abdomen
Digital Headbox	1	—	On the upper left chest, below the shoulder

Before placing the EEG electrodes, each marked point was thoroughly cleaned with the NuPrep gel. The gold cup electrodes were filled with the Ten20 conductive paste, and the EC2+ cream was used to secure them to the skin or hair. After placement, the connection quality of each electrode was verified using a *Checktrode Impedance Meter* [102]. If the impedance readings were satisfactory, the electrode was then connected to its corresponding position in the Digital Headbox. The EOG and EMG electrodes were placed similarly, as detailed in Table 1. However, instead of the EC2+ cream, surgical tape or (more skin-friendly) *Hypafix Retention Tape* [103] was used for fixation on the skin. Fixating the EMG electrodes was often challenging, especially for participants with short facial hair on their chins. If necessary, the reference EMG electrode was slightly shifted to the right of the chin’s midline, and the other two were moved below their intended positions where facial hair was less dense. These adjustments were carefully recorded to assist the sleep scorers in labeling the PSG data. Once all PSG electrodes were in place, the participant’s forehead was cleaned with rubbing alcohol. The Zmax headband, with its associated wet-EEG patch, was positioned slightly below the midline, right above the eyebrows. The experimental headband was placed on the remaining forehead space above the Zmax. The data quality of the experimental headband was briefly checked. The participants were asked if they were comfortable wearing the headbands. If not, the tightness of their straps was adjusted. The participants were also encouraged to adjust the straps if they felt any discomfort later on. Finally, the Empatica wristband and the experimental smartwatch were put on the wrists of the nondominant and dominant hands of the participant, respectively, after their contact points had been cleaned with rubbing alcohol.

After all devices were correctly positioned, participants were reminded of the instructions, which were also provided in written form as a checklist (see the Data Availability section for more details). Three of the six devices (Somnoscreen, Zmax, and the experimental headband) were not activated while the participants were in the lab due to their limited battery life. Participants were instructed and demonstrated how to turn these devices on and start recording before going to bed, as well as how to turn them off and stop recording the following morning. Given the importance of these steps, instructions were reiterated multiple times, including during the intake session, and participants practiced these actions in the lab to minimize the chances of failure. They were also provided with an accompanying video [104]. As these devices were activated at different times, synchronizing all device recordings posed a challenge, especially since some lacked a reliable internal clock, making straightforward time-based synchronization unreliable. Instead, we aimed for event-based synchronization. For this purpose, participants were instructed to perform a distinct activity (in our case, jumping five times) both before going to bed and upon waking up, which would be detectable across all six devices’ readings, particularly the accelerometers. We planned to use this activity to synchronize the devices’ recordings later on. Participants were shown how to jump while securing the devices with their hands effectively. Before leaving the lab, the experimenters went through another checklist to ensure that all preparatory steps were thoroughly completed.

Participants also filled in the mentioned questionnaires addressing their sleep, dreams, cognition, and overall health. Since the questions pertained to the participants’ cumulative experiences rather than any specific day, they had the flexibility to complete them any time after leaving the lab but within the day following their session. In addition, a customized questionnaire focused on the degree of the participants’ comfort (or discomfort) while wearing the devices at night. This questionnaire was completed the following morning, and the responses were used to adjust the preparation routine, enhancing comfort and the overall experience for subsequent participants.

If participants needed to use the restroom or leave the bed for other activities, they were instructed to do so normally, without turning off the devices, as we planned to detect (and exclude, if necessary) these periods later from the recording based on the devices’ accelerometer readings. The morning after the session, participants returned the devices at a prearranged place and time. Upon receipt, experimenters downloaded the data from all devices and conducted preliminary quality checks to assess the overall usability of the recordings and determine if any adjustments were necessary. The devices were then thoroughly cleaned and prepared for the following participants. Three to four sessions were conducted each week, typically on weekdays, resulting in data from at least five participants per week. Participants were also encouraged to report any adverse events they experienced during the project; these were promptly documented, and appropriate advice and support were provided.

### Data preprocessing

Here, we outline the preprocessing steps upon collecting the data. Our initial analysis encompasses both automatic and manual scoring of the PSG data, automatically identifying the Time in Bed (TIB) period from the Zmax data, synchronization across the four devices, and compiling a cleaned, time-synced version of the dataset, titled *Wearanize + PlugNPlay*, structured to facilitate ease of use across various computational environments.

#### Sleep-scoring PSG recordings

An experienced sleep scorer manually checked and sleep-scored the PSG data, which might serve as the ground truth for the majority of the applications. Manual scoring was done in 30-second epochs following the standard AASM sleep-scoring manual [4], along with arousal detection. Given the labor-intensive nature of the task, only nights with both successful PSG and Zmax recordings were manually scored. However, some nights were too noisy to score. In addition to manual scoring, the sleep recordings have also been classified using the *U-Sleep v2.0* [105] autoscoring algorithm. A comparison between the two sets of scores has been provided in the Sleep Scores Comparison section. Both sets are available in the dataset.

#### TIB detection from Zmax recordings

**Figure 3:**
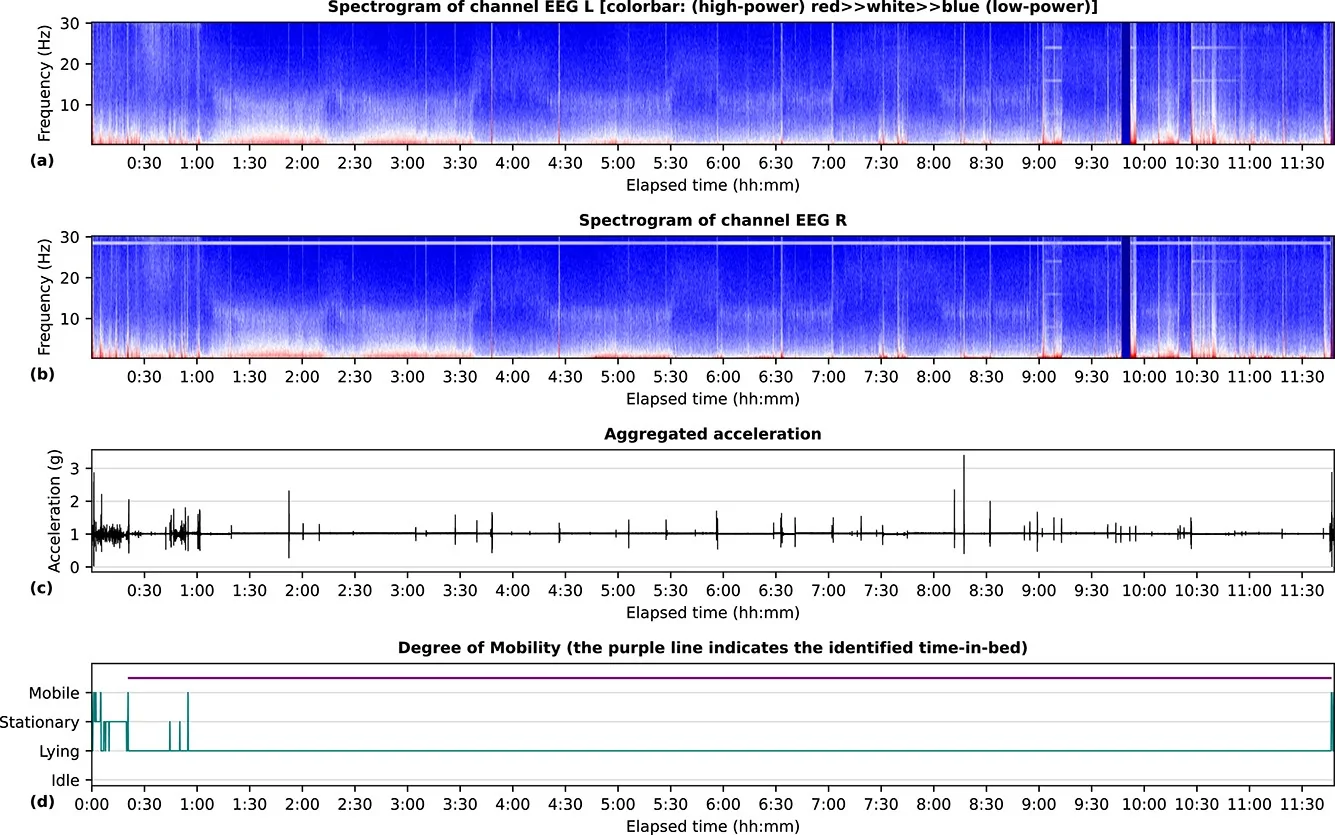
Visualization of a sample Zmax recording in terms of its (a) EEG-left channel spectrogram, (b) EEG-right channel spectrogram, (c) normalized accelerometer, and (d) the identified lying down period by eegMobility.

We used a machine learning model named *eegMobility* (a part of the *eegFloss* package [106]) to automatically detect a participant’s degree of mobility throughout the night based on tri-axial accelerometer readouts of the Zmax. The model was trained on a small dataset containing various activity data, including sleep, collected using Zmax [107]. It functions similarly to a Human Activity Recognition (HAR) model [108], but instead of classifying specific activities, it is designed to assess movement intensity. Specifically, it distinguishes between four states: lying down, slow movements (such as sitting down or standing up), moderate to fast movements (such as walking, jumping, or biking), and idle periods (when the device is not in use but still recording data), based solely on Zmax accelerometer readings. Based on its classification outputs, we pinpointed the moments a participant went to bed and woke up (also referred to as *Lights Out* and *Lights On* moments, respectively, and defined as the initial and final 2 minutes of continuous lying down during the night). The period between these two moments represents the participant’s TIB for the recorded night. Figure 3 illustrates the raw Zmax EEG data of a participant as spectrograms, the aggregated accelerometer output (described in the next section), and the mobility levels provided by eegMobility, along with the derived TIB. This automated TIB detection allows for the exclusion of nonsleep-related parts from the beginning and the end of the recording.

#### Synchronization

As outlined in the Data Collection Procedure section, we instructed participants to perform five jumps before bedtime and upon waking as a unique movement intended for synchronizing device recordings. However, considering our aim to develop applications demanding high precision, we later opted to manually synchronize each participant’s recordings through visual inspection of their corresponding raw tri-axial accelerometer data. As described in the TIB Detection from Zmax Recordings section, the Lights Out and Lights On moments were automatically determined from the Zmax data, and these moments also served as synchronization boundaries for the other three devices.

**Figure 4:**
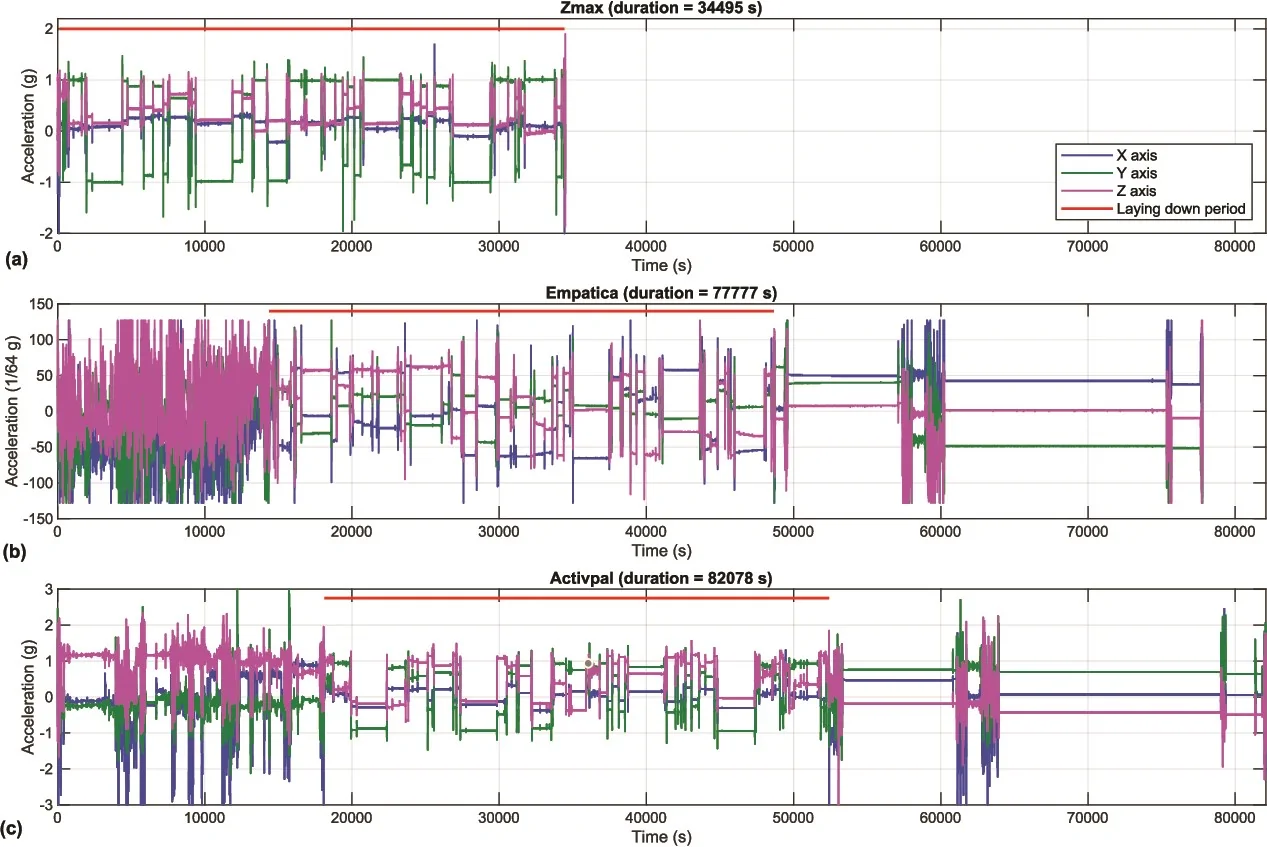
Manual synchronization dashboard of the (a) Zmax, (b) Empatica, and (c) Activpal recordings based on accelerometer data. The horizontal line at the top of each subfigure marks the desired lying period.

Both Empatica and Activpal were activated while participants were still in the lab and continued recording postawakening. Consequently, they had longer recording durations than Zmax. Figure 4 displays the dashboard used to visually synchronize the Empatica and Activpal recordings with Zmax using their tri-axial accelerometer outputs based on the visual identification of these common events. The targeted segments for synchronization are the defined sleep periods, bounded by the Lights Out and Lights On moments. Mentalab recordings were synchronized with their corresponding Zmax recordings following the same principles.

Somnoscreen, in contrast, does not retain the raw tri-axial accelerometer outputs but an aggregated form, indicating movement events (labeled as *Move.*). This aggregation complicates direct event comparison with the corresponding Zmax recordings. However, a similar aggregation of the Zmax tri-axial accelerometer data can be achieved using the following steps.

The tri-axial Zmax accelerometer signals can be represented as sequences ${\left({x}_t\right)}_{t=1,2,\dots, n}$, ${\left({y}_t\right)}_{t=1,2,\dots, n}$, and ${\left({z}_t\right)}_{t=1,2,\dots, n}$, corresponding to the *x*-, *y*-, and *z*-axes, respectively. For a given time-point *t*, the Euclidean norm (magnitude) of the acceleration can be calculated as:


(1) \begin{equation*} {a}_{\operatorname{norm},t}=\sqrt{x_t^2+{y}_t^2+{z}_t^2},\kern0.33em \mathrm{for}\kern0.33em t=1,2,\dots, n. \end{equation*}

This step aggregates the acceleration across the three axes into a single value, representing the magnitude of movement at each time point. To ensure all values are greater than or equal to 1, the following transformation was applied:


(2) \begin{equation*} {b}_{\operatorname{norm},t}=\left\{\begin{array}{ll}2-{a}_{\operatorname{norm},t},& \mathrm{if}\kern0.33em {a}_{\operatorname{norm},t}<1,\\{}{a}_{\operatorname{norm},t},& \mathrm{otherwise}.\end{array}\right. \end{equation*}

This transformation flips the values below 1 around the midpoint. Finally, the adjusted normalized values are shifted to center the data around 0 by subtracting 1 using:


(3) \begin{equation*} {c}_{\operatorname{norm},t}={b}_{\operatorname{norm},t}-1. \end{equation*}

**Figure 5:**
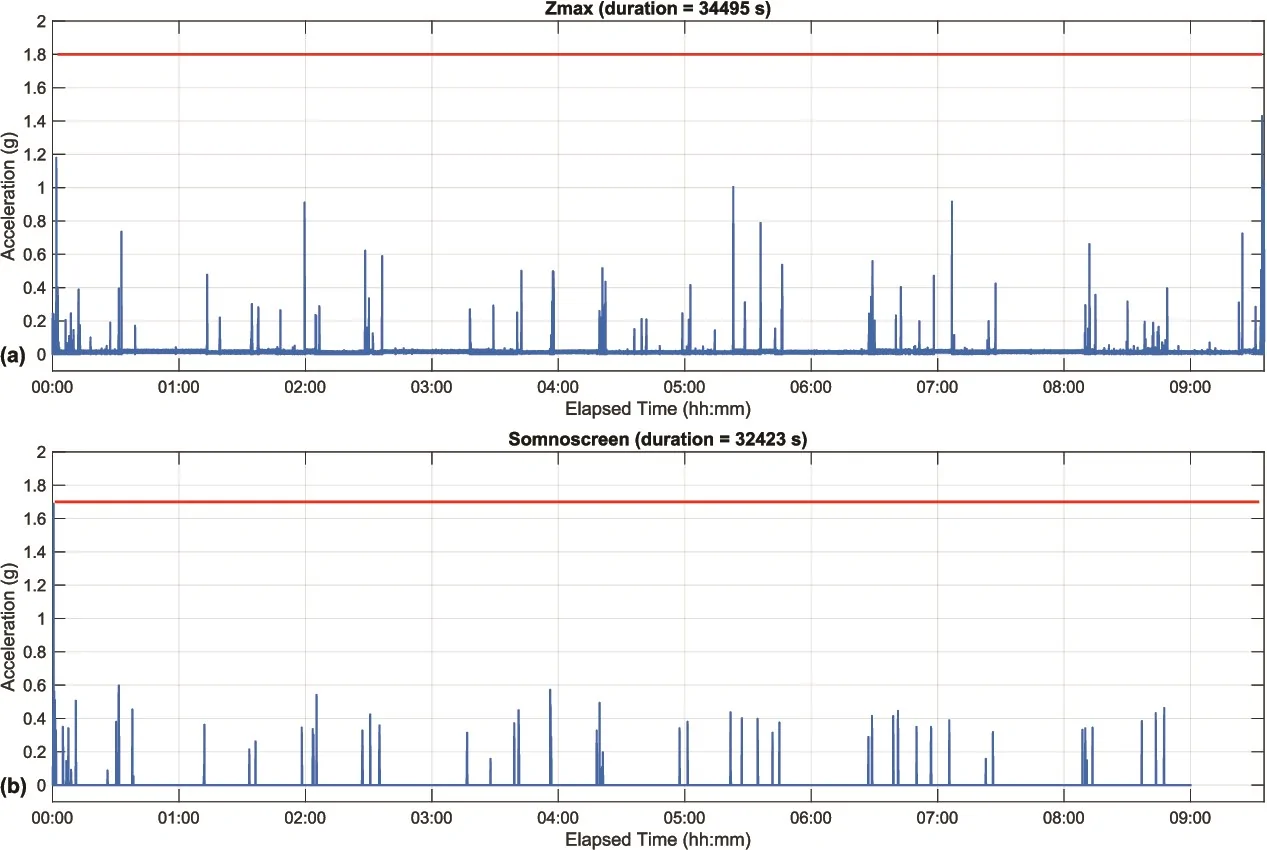
Manual synchronization dashboard of (a) Zmax and (b) Somnoscreen recordings based on their aggregated accelerometer data. The horizontal line at the top of each subfigure marks the identified lying period.

Following the steps outlined, the calculated ${c}_{norm}$ becomes visually analogous to the movement data from Somnoscreen, greatly simplifying synchronization using the previously described principles. Figure 5 illustrates the dashboard used for synchronizing Zmax and Somnoscreen recordings. As indicated in the figure, the duration of the Somnoscreen recording is, at times, shorter than that of the Zmax. This discrepancy stems from the limited battery life of Somnoscreen, an issue encountered with several subjects. In these cases, the durations of the Zmax, Empatica, and Activpal recordings were adjusted to ensure proper alignment with PSG recordings (and their associated sleep scores).

Later, we developed an automatic method for synchronization between Zmax and Somnoscreen based on cross-correlation. First, ${c}_{\mathit{\operatorname{norm}},t}$ was resampled to the frequency of Somnoscreen’s movement data; then, the resultant signal was rectified and centered using equations (2) and (3), followed by amplification and thresholding. Somnoscreen’s movement data are scaled, clipped, and thresholded similarly. Finally, cross-correlations were computed over nonnegative lags, and the lag with the maximum correlation was used to identify the best-aligned window. The full algorithm has been described in Appendix 2. However, since the outcome of the manual process was more reliable, this information has been included in the final dataset.

## Results

The primary outcome of the project is the Wearanize+ dataset. In this section, we quantitatively describe the data modalities recorded by each device and the output after initial quality checks. In total, we collected data from 130 participants aged 18–39 years (mean = 23.16 years, *SD* = 4.34, 89 females), with 116 participants being right-handed. This dataset includes PSG data from 118 participants, Zmax data from 122, Empatica data from 121, and Activpal data from 129, as well as the participants’ demographics and their responses to the three questionnaires (PSQI, MADRE, and PHQ-9). Recordings with very poor data quality were excluded. A summary of the participant-wise available recordings has been provided in Appendix 1. To ensure transparency and usability, we provide the Wearanize+ dataset in two sets: one containing the raw device data and the other containing a cleaned, time-synchronized version obtained upon applying the preprocessing steps described in the Data Preprocessing section.

### Wearanize+ raw dataset

The raw data are available for all 130 participants. However, not every device worked successfully for every participant. The exact number of recordings in the raw dataset is as follows: Somnoscreen, *n* = 103; Mentalab, *n* = 15; Zmax, *n* = 122; Empatica, *n* = 121; and Activpal, *n* = 129. Manual sleep scores are available for 103 of the 118 PSG recordings. The eegMobility-based TIB detection using eegFloss (described in the TIB Detection from Zmax Recordings section) was successful for 120 of the 122 Zmax recordings—the remaining two were adjusted manually. The manual synchronization (described in the Synchronization section) between Zmax, Empatica, Activpal, and Somnoscreen was successful for the majority of the participants. We have found shorter Somnoscreen recordings than Zmax in the case of around 29 participants, which were adjusted accordingly for the PlugNPlay version. Additional information on this set of information has been provided in Appendix 3.

### Wearanize+ PlugNPlay

To streamline usability and avoid repetition of the extensive preprocessing steps, the synchronized data from the four mentioned devices for each participant were consolidated into a single EDF file, while preserving all metadata and signal properties. Manual and automatic sleep scores derived from PSG were also integrated into the EDF files at a sampling rate of *1/30* Hz. Time-series signals were labeled according to the convention *[device_name]_[channel_name]*. This curated collection, referred to as the PlugNPlay version, includes data from 100 participants (out of the total 130; see Appendix 1 for details) for whom both PSG and Zmax data were available and manual sleep scoring could be performed. For some participants, Empatica and ActivPAL recordings are absent, either due to unsuccessful acquisition or issues with reliable synchronization. The PlugNPlay version is formatted according to the EEG-Brain Imaging Data Structure (EEG-BIDS) v1.10.0 specifications [109, 110]. The usability of the EEG signals has been checked with eegFloss, and the outputs are a part of this version as well. Further details are provided in the accompanying *README* and *dataset_description* files within the dataset.

**Figure 6:**
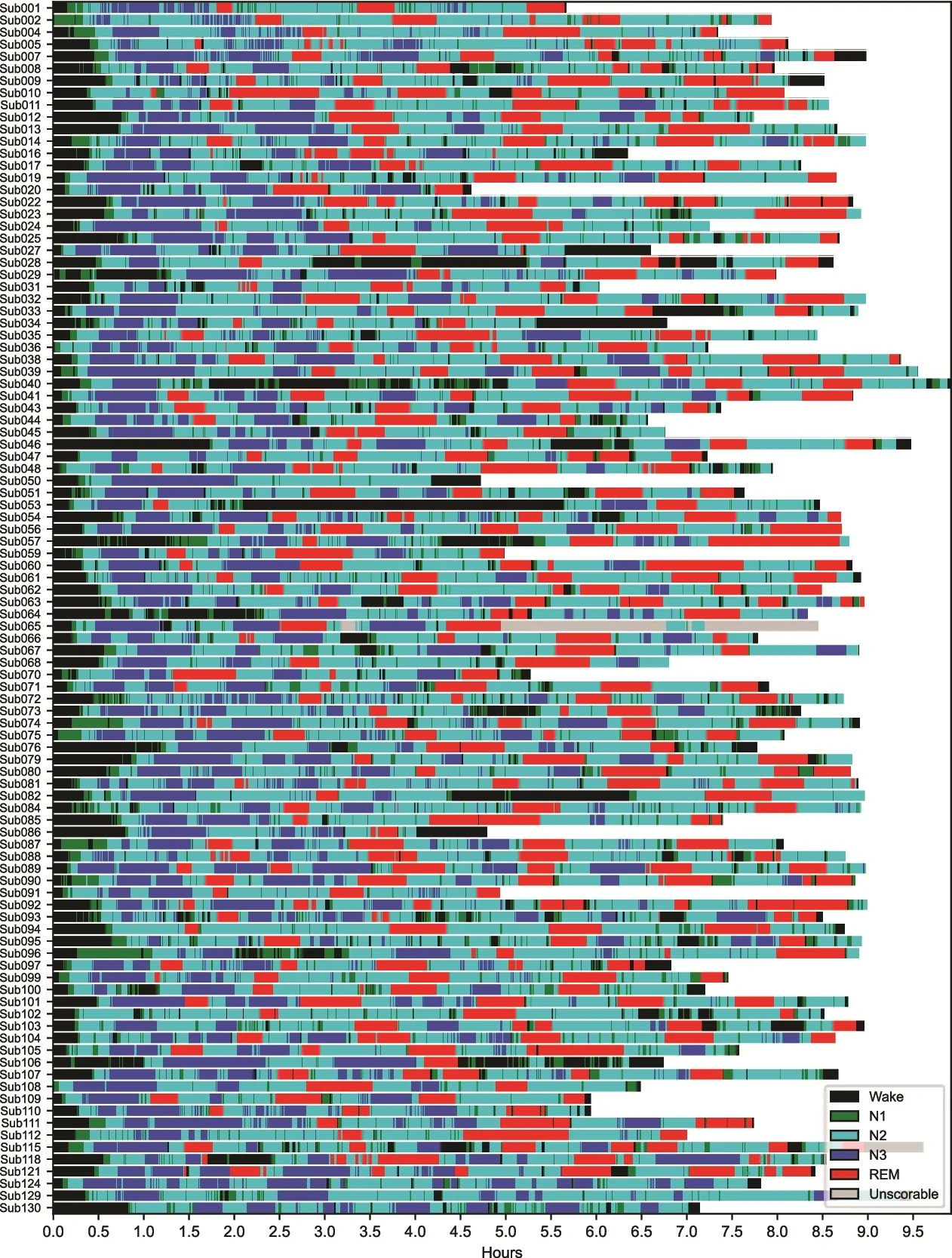
A linear representation of the participants’ sleep stages manually identified from their PSG recordings.

Figure 6 presents a stacked hypnogram detailing the manually identified sleep stages of the participants present in the PlugNPlay dataset. As depicted, the TIB for participants ranged from approximately 4.5–10 hours, with a median close to 8.5 hours, indicating that most participants achieved adequate sleep on the recorded night. The general rule of having more deep sleep (N3) during the first half of the night and more rapid eye movement (REM) sleep during the second also applies to most participants [111].

### Sleep scores comparison

**Figure 7:**
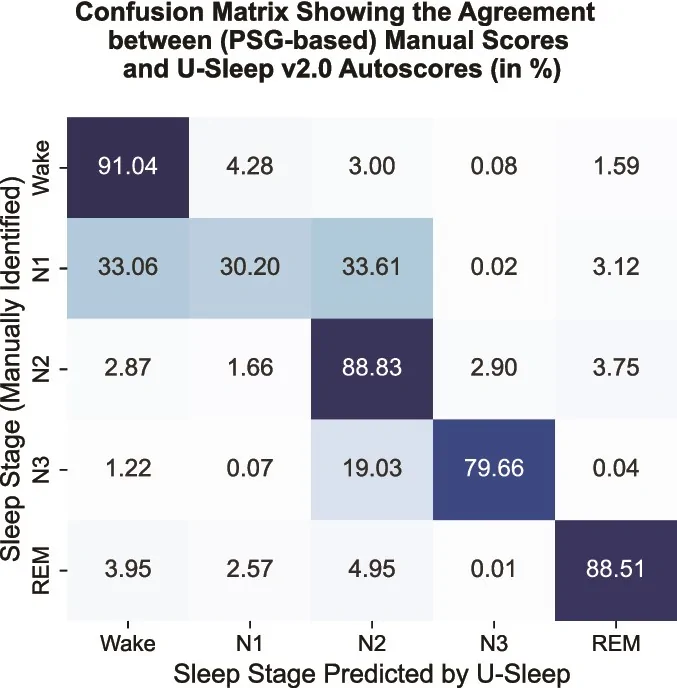
Comparison between manual sleep scores and autoscores provided by U-Sleep v2.0 on PSG data.

As mentioned earlier, 103 of the 118 PSG recordings were manually scored by an experienced sleep scorer. Somnoscreen recordings were also automatically scored by the U-Sleep v2.0 algorithm. In Figure 7, we present a confusion matrix comparing the two sets of scores of 96 participants. Overall, they show substantial agreement (Kappa = 0.769, F1 score = 0.836), which is evident from the presented confusion matrix.

## Discussion

In this paper, we describe the curation and the contents of the Wearanize+ dataset—a multimodal dataset containing sleep data of 130 healthy participants (one night each) recorded simultaneously using five wearables (three currently available) and traditional PSG. Moreover, it contains the participants’ responses to three questionnaires that provide information on their sleep, dreams, mood, and stress. This parallel setup makes the dataset particularly interesting and useful for various applications. Among the 130 participants, PSG data from 118, Zmax data from 122, Empatica data from 121, and Activpal data from 129 are available. The PSG data were manually sleep-scored, and the recordings were manually synchronized based on their corresponding accelerometer outputs. We observed a substantial agreement between manual sleep scores and autoscores (by U-Sleep v2.0) derived from the PSG recordings.

The primary limitation of the dataset is its limited intraparticipant variability, since each participant contributed a single night of recording, which may hinder testing hypotheses that require within-subject or longitudinal data, such as night-to-night variability and first-night effects. The dataset may also be limited in terms of interparticipant variability for some studies, particularly those focused on different age groups or on sleep and other disorders, as most of its data come from healthy young adult participants. The key challenges we faced during data collection included dealing with the limited battery life of some devices (which led to data loss) and inaccuracies in some devices’ internal clocks, necessitating manual synchronization of the recordings. The dataset is available to be used for research purposes (see the Data Availability section). For ease of use, a fully synchronized, processed, and curated version of the dataset, along with the accompanying sleep stages, has been provided in addition to the raw data.

This dataset can facilitate a range of applications, including device-specific validations of the three wearables, development of (device-specific) autoscorers based on PSG ground truths, methods for handling missing or corrupted data, and evaluation of alternative (as well as compound) sensor modalities for sleep scoring. It has already supported the development of Zmax autoscorers, such as *ezscore-f* by Coon et al. [112]. It has also facilitated the validation of our automatic Zmax–Somnoscreen synchronization method. One of our key objectives is to leverage these multimodal recordings to build a robust, multiwearable sleep-scoring model that approaches PSG-grade performance while minimizing the impact of EEG artifacts, with direct benefits for the larger, wearable-based HBS dataset. We hope that other researchers will also find the dataset useful for their research and incorporate it into their projects.

## Supplementary Material

Wearanize_Supplementary_Materials_zpaf094

## Data Availability

The Wearanize+ dataset is hosted on the Radboud Data Repository at doi.org/10.34973/j6jf-9e62 and is available for use in scientific research upon signing a Data Use Agreement. The procedure for accessing the dataset is outlined at GitHub.com/Niloy333/Wearanize_plus, which also includes the scripts used for data preprocessing. The email invitation, informed consent form, checklists, session procedure, and other related documents are also available in the GitHub repository.
