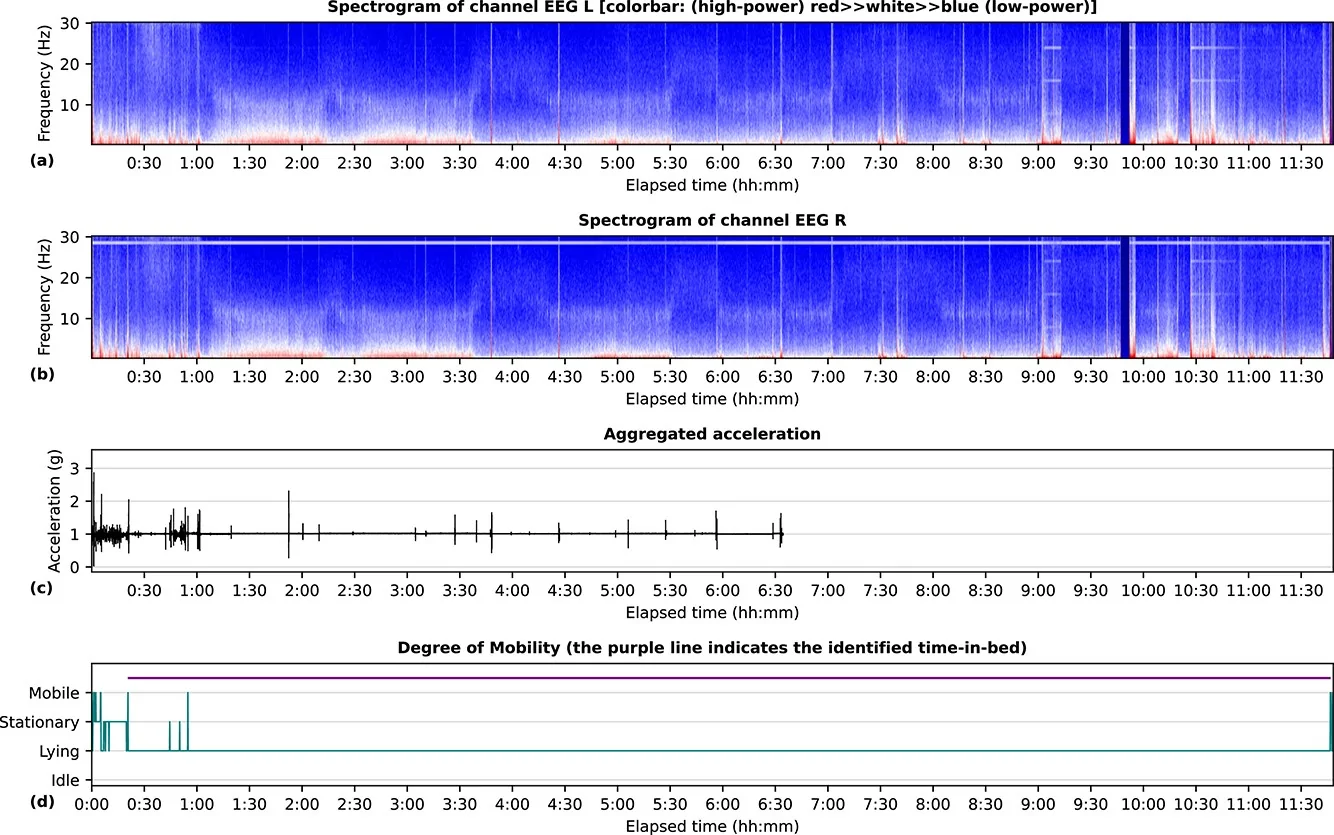# Correction to: *Wearanize+*: a multimodal dataset for evaluating wearable technologies in sleep research

**DOI:** 10.1093/sleepadvances/zpag104

**Published:** 2026-10-13

This is a correction to: Niloy Sikder, Lieuwe Verkaar, Anastasiya Paltarzhytskaya, Selin Acan, Leonore Bovy, Tatiana Almazova, Elena Krugliakova, Yevgenia Rosenblum, Matthias Krauledat, Martin Dresler, Paul Zerr, *Wearanize+*: a multimodal dataset for evaluating wearable technologies in sleep research, *SLEEP Advances*, Volume 7, Issue 1, 2026, zpaf094, https://doi.org/10.1093/sleepadvances/zpaf094

In the originally published version of this manuscript, subplot C within Figure 3 did not display the entire line for the full duration of the recording.

This has been corrected so that Figure 3 reads:


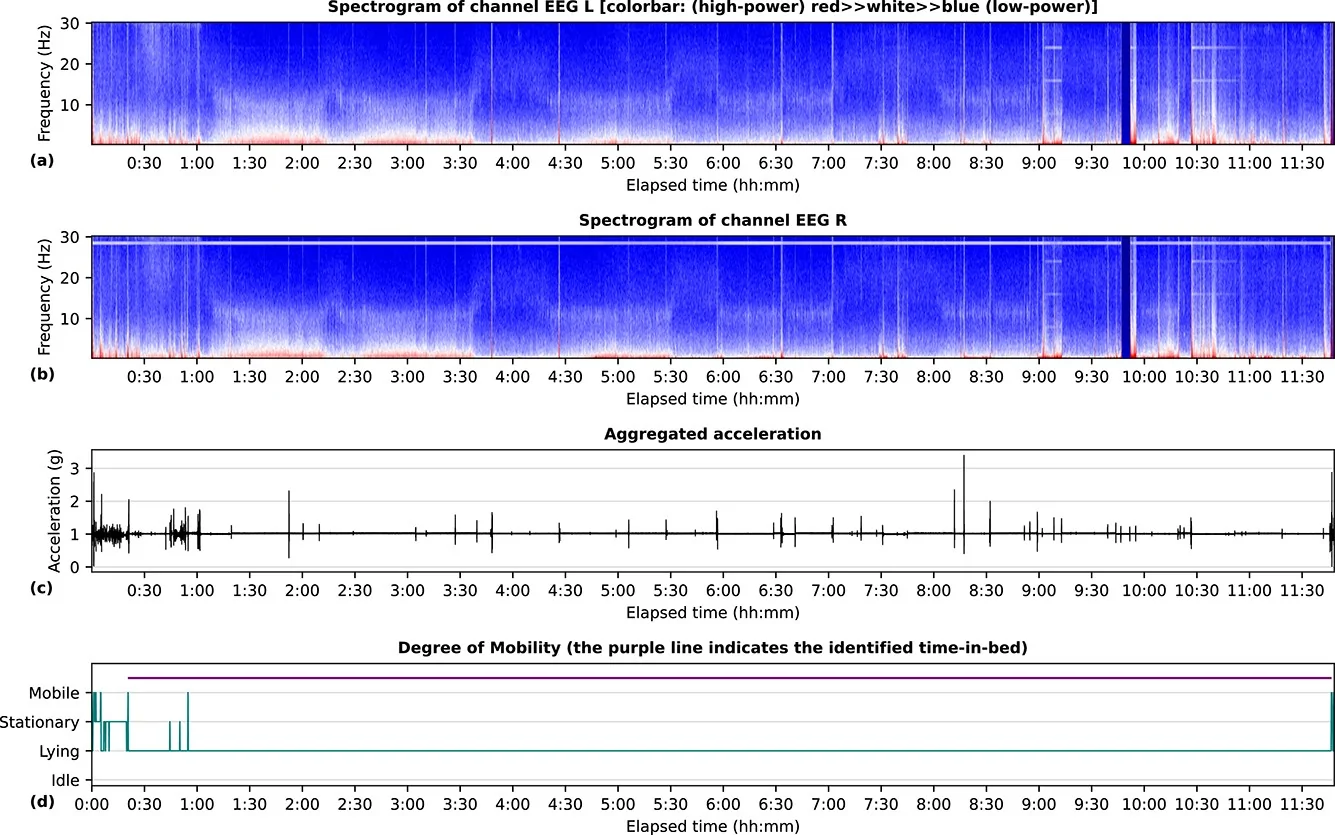


Instead of: