# Supplementary material for: Wearanize+: a multimodal dataset for evaluating wearable technologies in sleep research
Source: Sleep Adv. 2025 Dec 27;7(1):zpaf094. doi: 10.1093/sleepadvances/zpaf094 (PMC12888818; doi:10.1093/sleepadvances/zpaf094)
Supplement: Wearanize_Supplementary_Materials_zpaf094 [file wearanize_supplementary_materials_zpaf094.docx]

**Wearanize+: A Multimodal Dataset for Evaluating Wearable Technologies in Sleep Research [Supplementary Materials]**

##

## Appendix 1. Participant-wise Data Availability

**Table 1.1:** Participant and device-wise data availability after preliminary assessment.

| **Subject ID** | **PSG** | **Manual Scores** | **Zmax** | **Empatica** | **Activpal** | **PlugNPlay** | **PSQI** | **MADRE** | **PHQ-9** |
| --- | --- | --- | --- | --- | --- | --- | --- | --- | --- |
| Sub001 | ✓ | ✓ | ✓ | ✕ | ✓ | ✓ | ✓ | ✓ | ✓ |
| Sub002 | ✓ | ✓ | ✓ | ✕ | ✓ | ✓ | ✓ | ✓ | ✓ |
| Sub003 | ✓ | ✕ | ✕ | ✕ | ✓ | ✕ | ✓ | ✓ | ✓ |
| Sub004 | ✓ | ✓ | ✓ | ✕ | ✓ | ✓ | ✓ | ✓ | ✓ |
| Sub005 | ✓ | ✓ | ✓ | ✓ | ✓ | ✓ | ✓ | ✓ | ✓ |
| Sub006 | ✕ | ✕ | ✕ | ✓ | ✓ | ✕ | ✓ | ✓ | ✓ |
| Sub007 | ✓ | ✓ | ✓ | ✓ | ✓ | ✓ | ✓ | ✓ | ✓ |
| Sub008 | ✓ | ✓ | ✓ | ✓ | ✓ | ✓ | ✓ | ✓ | ✓ |
| Sub009 | ✓ | ✓ | ✓ | ✓ | ✓ | ✓ | ✓ | ✓ | ✓ |
| Sub010 | ✓ | ✓ | ✓ | ✓ | ✓ | ✓ | ✓ | ✓ | ✓ |
| Sub011 | ✓ | ✓ | ✓ | ✓ | ✓ | ✓ | ✓ | ✓ | ✓ |
| Sub012 | ✓ | ✓ | ✓ | ✓ | ✓ | ✓ | ✓ | ✓ | ✓ |
| Sub013 | ✓ | ✓ | ✓ | ✓ | ✓ | ✓ | ✓ | ✓ | ✓ |
| Sub014 | ✓ | ✓ | ✓ | ✓ | ✓ | ✓ | ✓ | ✓ | ✓ |
| Sub015 | ✕ | ✕ | ✓ | ✓ | ✓ | ✕ | ✓ | ✓ | ✓ |
| Sub016 | ✓ | ✓ | ✓ | ✓ | ✓ | ✓ | ✓ | ✓ | ✓ |
| Sub017 | ✓ | ✓ | ✓ | ✓ | ✓ | ✓ | ✓ | ✓ | ✓ |
| Sub018 | ✕ | ✕ | ✓ | ✓ | ✓ | ✕ | ✓ | ✓ | ✓ |
| Sub019 | ✓ | ✓ | ✓ | ✓ | ✓ | ✓ | ✓ | ✓ | ✓ |
| Sub020 | ✓ | ✓ | ✓ | ✓ | ✓ | ✓ | ✓ | ✓ | ✓ |
| Sub021 | ✕ | ✕ | ✓ | ✓ | ✓ | ✕ | ✓ | ✓ | ✓ |
| Sub022 | ✓ | ✓ | ✓ | ✓ | ✓ | ✓ | ✓ | ✓ | ✓ |
| Sub023 | ✓ | ✓ | ✓ | ✓ | ✓ | ✓ | ✓ | ✓ | ✓ |
| Sub024 | ✓ | ✓ | ✓ | ✓ | ✓ | ✓ | ✓ | ✓ | ✓ |
| Sub025 | ✓ | ✓ | ✓ | ✓ | ✓ | ✓ | ✓ | ✓ | ✓ |
| Sub026 | ✕ | ✕ | ✓ | ✓ | ✓ | ✕ | ✓ | ✓ | ✓ |
| Sub027 | ✓ | ✓ | ✓ | ✓ | ✓ | ✓ | ✓ | ✓ | ✓ |
| Sub028 | ✓ | ✓ | ✓ | ✓ | ✓ | ✓ | ✓ | ✓ | ✓ |
| Sub029 | ✓ | ✓ | ✓ | ✓ | ✓ | ✓ | ✓ | ✓ | ✓ |
| Sub030 | ✕ | ✕ | ✕ | ✓ | ✓ | ✕ | ✓ | ✓ | ✓ |
| Sub031 | ✓ | ✓ | ✓ | ✓ | ✓ | ✓ | ✓ | ✓ | ✓ |
| Sub032 | ✓ | ✓ | ✓ | ✓ | ✓ | ✓ | ✓* | ✓* | ✓* |
| Sub033 | ✓ | ✓ | ✓ | ✓ | ✓ | ✓ | ✓ | ✓ | ✓ |
| Sub034 | ✓ | ✓ | ✓ | ✓ | ✓ | ✓ | ✓ | ✓ | ✓ |
| Sub035 | ✓ | ✓ | ✓ | ✓ | ✓ | ✓ | ✓ | ✓ | ✓ |
| Sub036 | ✓ | ✓ | ✓ | ✓ | ✓ | ✓ | ✓ | ✓ | ✓ |
| Sub037 | ✓ | ✕ | ✓ | ✓ | ✓ | ✕ | ✓ | ✓ | ✓ |
| Sub038 | ✓ | ✓ | ✓ | ✓ | ✓ | ✓ | ✓ | ✓ | ✓ |
| Sub039 | ✓ | ✓ | ✓ | ✓ | ✓ | ✓ | ✓ | ✓ | ✓ |
| Sub040 | ✓ | ✓ | ✓ | ✓ | ✓ | ✓ | ✓ | ✓ | ✓ |
| Sub041 | ✓ | ✓ | ✓ | ✓ | ✓ | ✓ | ✓ | ✓ | ✓ |
| Sub042 | ✕ | ✕ | ✓ | ✓ | ✓ | ✕ | ✓ | ✓ | ✓ |
| Sub043 | ✓ | ✓ | ✓ | ✓ | ✓ | ✓ | ✓ | ✓ | ✓ |
| Sub044 | ✓ | ✓ | ✓ | ✓ | ✓ | ✓ | ✓ | ✓ | ✓ |
| Sub045 | ✓ | ✓ | ✓ | ✓ | ✓ | ✓ | ✓ | ✓ | ✓ |
| Sub046 | ✓ | ✓ | ✓ | ✓ | ✓ | ✓ | ✓ | ✓ | ✓ |
| Sub047 | ✓ | ✓ | ✓ | ✓ | ✓ | ✓ | ✓ | ✓ | ✓ |
| Sub048 | ✓ | ✓ | ✓ | ✓ | ✓ | ✓ | ✓ | ✓ | ✓ |
| Sub049 | ✕ | ✕ | ✓ | ✓ | ✓ | ✕ | ✓ | ✓ | ✓ |
| Sub050 | ✓ | ✓ | ✓ | ✓ | ✓ | ✓ | ✓ | ✓ | ✓ |
| Sub051 | ✓ | ✓ | ✓ | ✓ | ✓ | ✓ | ✓ | ✓ | ✓ |
| Sub052 | ✕ | ✕ | ✓ | ✓ | ✓ | ✕ | ✓ | ✓ | ✓ |
| Sub053 | ✓ | ✓ | ✓ | ✓ | ✓ | ✓ | ✓ | ✓ | ✓ |
| Sub054 | ✓ | ✓ | ✓ | ✓ | ✓ | ✓ | ✓ | ✓ | ✓ |
| Sub055 | ✕ | ✕ | ✓ | ✓ | ✓ | ✕ | ✓ | ✓ | ✓ |
| Sub056 | ✓ | ✓ | ✓ | ✓ | ✓ | ✓ | ✓ | ✓ | ✓ |
| Sub057 | ✓ | ✓ | ✓ | ✓ | ✓ | ✓ | ✓ | ✓ | ✓ |
| Sub058 | ✕ | ✕ | ✓ | ✓ | ✓ | ✕ | ✓ | ✓ | ✓ |
| Sub059 | ✓ | ✓ | ✓ | ✓ | ✓ | ✓ | ✓ | ✓ | ✓ |
| Sub060 | ✓ | ✓ | ✓ | ✓ | ✓ | ✓ | ✓ | ✓ | ✓ |
| Sub061 | ✓ | ✓ | ✓ | ✓ | ✓ | ✓ | ✓ | ✓ | ✓ |
| Sub062 | ✓ | ✓ | ✓ | ✓ | ✓ | ✓ | ✓ | ✓ | ✓ |
| Sub063 | ✓ | ✓ | ✓ | ✓ | ✓ | ✓ | ✓ | ✓ | ✓ |
| Sub064 | ✓ | ✓ | ✓ | ✓ | ✓ | ✓ | ✓ | ✓ | ✓ |
| Sub065 | ✓ | ✓ | ✓ | ✓ | ✓ | ✓ | ✓ | ✓ | ✓ |
| Sub066 | ✓ | ✓ | ✓ | ✓ | ✓ | ✓ | ✓ | ✓ | ✓ |
| Sub067 | ✓ | ✓ | ✓ | ✓ | ✓ | ✓ | ✓ | ✓ | ✓ |
| Sub068 | ✓ | ✓ | ✓ | ✓ | ✓ | ✓ | ✓ | ✓ | ✓ |
| Sub069 | ✓ | ✕ | ✕ | ✓ | ✓ | ✕ | ✓ | ✓ | ✓ |
| Sub070 | ✓ | ✓ | ✓ | ✓ | ✓ | ✓ | ✓ | ✓ | ✓ |
| Sub071 | ✓ | ✓ | ✓ | ✓ | ✓ | ✓ | ✓ | ✓ | ✓ |
| Sub072 | ✓ | ✓ | ✓ | ✓ | ✓ | ✓ | ✓ | ✓ | ✓ |
| Sub073 | ✓ | ✓ | ✓ | ✓ | ✓ | ✓ | ✓ | ✓ | ✓ |
| Sub074 | ✓ | ✓ | ✓ | ✓ | ✓ | ✓ | ✓ | ✓ | ✓ |
| Sub075 | ✓ | ✓ | ✓ | ✓ | ✓ | ✓ | ✓ | ✓ | ✓ |
| Sub076 | ✓ | ✓ | ✓ | ✓ | ✓ | ✓ | ✓ | ✓ | ✓ |
| Sub077 | ✓ | ✓ | ✕ | ✓ | ✓ | ✕ | ✓ | ✓ | ✓ |
| Sub078 | ✓ | ✓ | ✕ | ✓ | ✓ | ✕ | ✓ | ✓ | ✓ |
| Sub079 | ✓ | ✓ | ✓ | ✓ | ✓ | ✓ | ✓ | ✓ | ✓ |
| Sub080 | ✓ | ✓ | ✓ | ✓ | ✓ | ✓ | ✓ | ✓ | ✓ |
| Sub081 | ✓ | ✓ | ✓ | ✓ | ✓ | ✓ | ✓ | ✓ | ✓ |
| Sub082 | ✓ | ✓ | ✓ | ✓ | ✓ | ✓ | ✓ | ✓ | ✓ |
| Sub083 | ✓ | ✕ | ✓ | ✓ | ✓ | ✕ | ✓ | ✓ | ✓ |
| Sub084 | ✓ | ✓ | ✓ | ✓ | ✓ | ✓ | ✓ | ✓ | ✓ |
| Sub085 | ✓ | ✓ | ✓ | ✓ | ✓ | ✓ | ✓ | ✓ | ✓ |
| Sub086 | ✓ | ✓ | ✓ | ✓ | ✓ | ✓ | ✓ | ✓ | ✓ |
| Sub087 | ✓ | ✓ | ✓ | ✓ | ✓ | ✓ | ✓ | ✓ | ✓ |
| Sub088 | ✓ | ✓ | ✓ | ✓ | ✓ | ✓ | ✓ | ✓ | ✓ |
| Sub089 | ✓ | ✓ | ✓ | ✓ | ✓ | ✓ | ✓ | ✓ | ✓ |
| Sub090 | ✓ | ✓ | ✓ | ✓ | ✓ | ✓ | ✓ | ✓ | ✓ |
| Sub091 | ✓ | ✓ | ✓ | ✓ | ✓ | ✓ | ✓ | ✓ | ✓ |
| Sub092 | ✓ | ✓ | ✓ | ✓ | ✓ | ✓ | ✓ | ✓ | ✓ |
| Sub093 | ✓ | ✓ | ✓ | ✓ | ✓ | ✓ | ✓ | ✓ | ✓ |
| Sub094 | ✓ | ✓ | ✓ | ✓ | ✓ | ✓ | ✓ | ✓ | ✓ |
| Sub095 | ✓ | ✓ | ✓ | ✓ | ✓ | ✓ | ✓ | ✓ | ✓ |
| Sub096 | ✓ | ✓ | ✓ | ✓ | ✓ | ✓ | ✓ | ✓ | ✓ |
| Sub097 | ✓ | ✓ | ✓ | ✓ | ✓ | ✓ | ✓ | ✓ | ✓ |
| Sub098 | ✓ | ✓ | ✕ | ✓ | ✓ | ✕ | ✓ | ✓ | ✓ |
| Sub099 | ✓ | ✓ | ✓ | ✓ | ✓ | ✓ | ✓ | ✓ | ✓ |
| Sub100 | ✓ | ✓ | ✓ | ✓ | ✓ | ✓ | ✓ | ✓ | ✓ |
| Sub101 | ✓ | ✓ | ✓ | ✓ | ✓ | ✓ | ✓ | ✓ | ✓ |
| Sub102 | ✓ | ✓ | ✓ | ✓ | ✓ | ✓ | ✓ | ✓ | ✓ |
| Sub103 | ✓ | ✓ | ✓ | ✓ | ✓ | ✓ | ✓ | ✓ | ✓ |
| Sub104 | ✓ | ✓ | ✓ | ✓ | ✓ | ✓ | ✓ | ✓ | ✓ |
| Sub105 | ✓ | ✓ | ✓ | ✓ | ✓ | ✓ | ✓ | ✓ | ✓ |
| Sub106 | ✓ | ✓ | ✓ | ✓ | ✓ | ✓ | ✓ | ✓ | ✓ |
| Sub107 | ✓ | ✓ | ✓ | ✓ | ✓ | ✓ | ✓ | ✓ | ✓ |
| Sub108 | ✓ | ✓ | ✓ | ✓ | ✓ | ✓ | ✓ | ✓ | ✓ |
| Sub109 | ✓ | ✓ | ✓ | ✓ | ✓ | ✓ | ✓ | ✓ | ✓ |
| Sub110 | ✓ | ✓ | ✓ | ✓ | ✓ | ✓ | ✓ | ✓ | ✓ |
| Sub111 | ✓ | ✓ | ✓ | ✓ | ✓ | ✓ | ✓ | ✓ | ✓ |
| Sub112 | ✓ | ✓ | ✓ | ✓ | ✓ | ✓ | ✓ | ✓ | ✓ |
| Sub113 | ✓^m^ | ✕ | ✓ | ✓ | ✓ | ✕ | ✓ | ✓ | ✓ |
| Sub114 | ✓^m^ | ✕ | ✓ | ✓ | ✓ | ✕ | ✓ | ✓ | ✓ |
| Sub115 | ✓^m^ | ✓ | ✓ | ✓ | ✓ | ✓ | ✓ | ✓ | ✓ |
| Sub116 | ✓^m^ | ✕ | ✓ | ✓ | ✓ | ✕ | ✓ | ✓ | ✓ |
| Sub117 | ✓^m^ | ✕ | ✓ | ✓ | ✓ | ✕ | ✓ | ✓ | ✓ |
| Sub118 | ✓ | ✓ | ✓ | ✓ | ✓ | ✓ | ✓ | ✓ | ✓ |
| Sub119 | ✓^m^ | ✕ | ✓ | ✓ | ✓ | ✕ | ✓ | ✓ | ✓ |
| Sub120 | ✓^m^ | ✕ | ✕ | ✓ | ✓ | ✕ | ✓ | ✓ | ✓ |
| Sub121 | ✓ | ✓ | ✓ | ✓ | ✓ | ✓ | ✓ | ✓ | ✓ |
| Sub122 | ✓^m^ | ✕ | ✓ | ✕ | ✕ | ✕ | ✓ | ✓ | ✓ |
| Sub123 | ✓^m^ | ✕ | ✓ | ✓ | ✓ | ✕ | ✓ | ✓ | ✓ |
| Sub124 | ✓^m^ | ✓ | ✓ | ✓ | ✓ | ✓ | ✓ | ✓ | ✓ |
| Sub125 | ✕ | ✕ | ✓ | ✓ | ✓ | ✕ | ✓ | ✓ | ✓ |
| Sub126 | ✓^m^ | ✕ | ✓ | ✕ | ✓ | ✕ | ✓ | ✓ | ✓ |
| Sub127 | ✓^m^ | ✕ | ✓ | ✓ | ✓ | ✕ | ✓ | ✓ | ✓ |
| Sub128 | ✓^m^ | ✕ | ✓ | ✕ | ✓ | ✕ | ✓ | ✓ | ✓ |
| Sub129 | ✓^m^ | ✓ | ✓ | ✕ | ✓ | ✓ | ✓ | ✓ | ✓ |
| Sub130 | ✓^m^ | ✓ | ✓ | ✕ | ✓ | ✓ | ✓ | ✓ | ✓ |
| **Total** | **118** | **103** | **122** | **121** | **129** | **100** | **130** | **130** | **130** |
| ✓: available, ✕: unavailable, ✓^m^: recorded with Mentalab ✓*: the questionnaire was partially completed | | | | | | | | | |

## Appendix 2. Zmax–Somnoscreen Automatic Synchronization Method

Algorithm 2.1 describes the automatic synchronization method for parallel Zmax and Somnoscreen recordings that we devised based on cross-correlations of the raw accelerometer signals of Zmax and the Move. signal of Somnoscreen. We have also provided a MATLAB script to apply the algorithm to new data in the associated GitHub repository.

| **Algorithm 2.1:** An algorithm to automatically synchronize parallel Zmax and Somnoscreen recordings | | |
| --- | --- | --- |
| **Inputs** | : | Tri-axial Zmax accelerometer signals [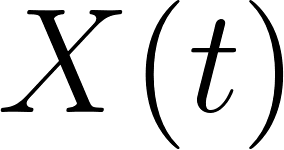](https://www.codecogs.com/eqnedit.php?latex=X(t)#0), [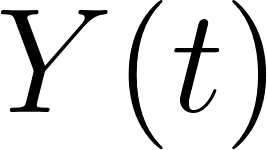](https://www.codecogs.com/eqnedit.php?latex=Y(t)#0), and [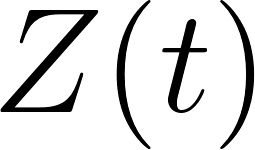](https://www.codecogs.com/eqnedit.php?latex=Z(t)#0), sampled at [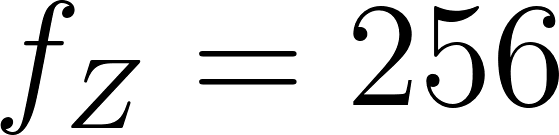](https://www.codecogs.com/eqnedit.php?latex=f_Z%3D256#0) Hz, Somnoscreen Move. signal [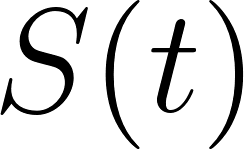](https://www.codecogs.com/eqnedit.php?latex=S(t)#0) sampled at [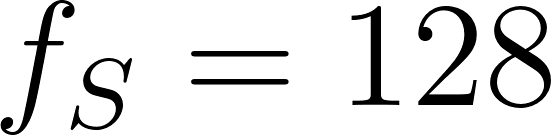](https://www.codecogs.com/eqnedit.php?latex=f_S%3D128#0) Hz, and The Lights Out and Lights On moments [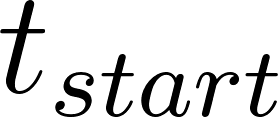](https://www.codecogs.com/eqnedit.php?latex=t_%7Bstart%7D#0) and [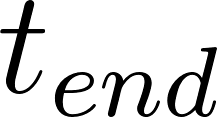](https://www.codecogs.com/eqnedit.php?latex=t_%7Bend%7D#0) detected from Zmax (in seconds). |
| **Output** | : | Estimated Lights Out moment [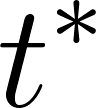](https://www.codecogs.com/eqnedit.php?latex=t%5E*#0) of Somnoscreen |
| Step-1 | : | Extract signal segments from Zmax within [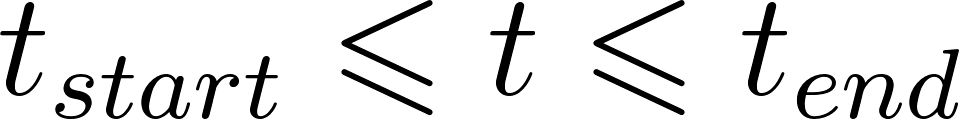](https://www.codecogs.com/eqnedit.php?latex=t_%7Bstart%7D%5Cleqslant%20t%5Cleqslant%20t_%7Bend%7D#0):  Let: [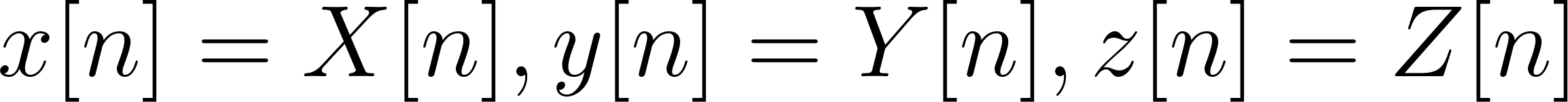](https://www.codecogs.com/eqnedit.php?latex=x%5Bn%5D%3DX%5Bn%5D%2C%20y%5Bn%5D%3DY%5Bn%5D%2C%20z%5Bn%5D%3DZ%5Bn%5D#0) where [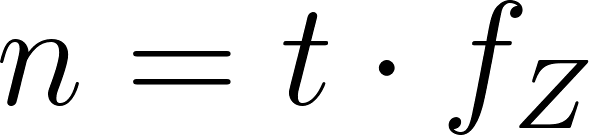](https://www.codecogs.com/eqnedit.php?latex=n%3Dt%5Ccdot%20f_Z#0) |
| Step-2 | : | Compute Euclidean norm of the Zmax signals:  [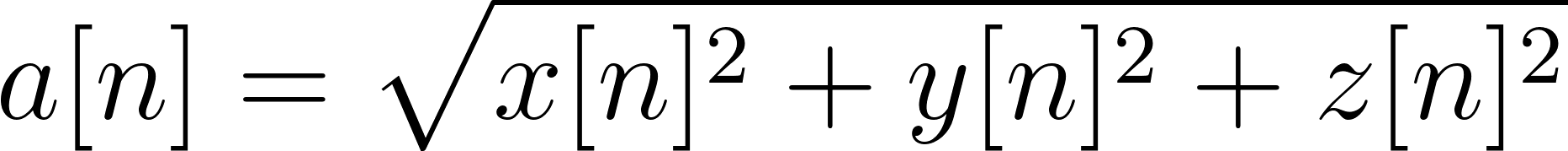](https://www.codecogs.com/eqnedit.php?latex=a%5Bn%5D%3D%5Csqrt%7Bx%5Bn%5D%5E2%2By%5Bn%5D%5E2%2Bz%5Bn%5D%5E2%7D#0) |
| Step-3 | : | Resample [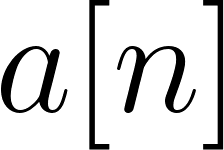](https://www.codecogs.com/eqnedit.php?latex=a%5Bn%5D#0) from [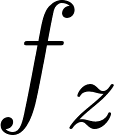](https://www.codecogs.com/eqnedit.php?latex=f_z#0) to [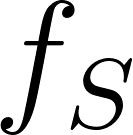](https://www.codecogs.com/eqnedit.php?latex=f_S#0) using shape preserving interpolation:  Let [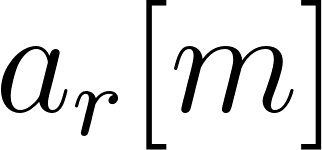](https://www.codecogs.com/eqnedit.php?latex=a_r%5Bm%5D#0) be the resampled signal where [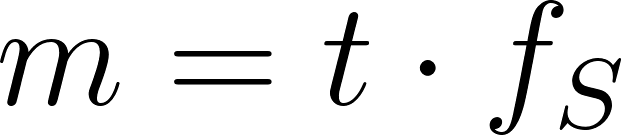](https://www.codecogs.com/eqnedit.php?latex=m%3Dt%5Ccdot%20f_S#0) |
| Step-4 | : | Rectify, center, and amplify [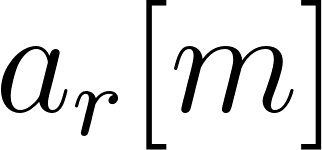](https://www.codecogs.com/eqnedit.php?latex=a_r%5Bm%5D#0):  If [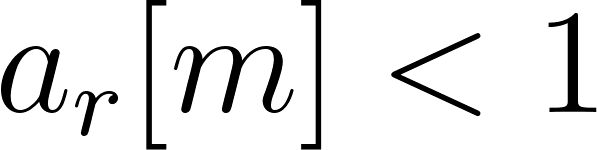](https://www.codecogs.com/eqnedit.php?latex=a_r%5Bm%5D%3C1#0), set [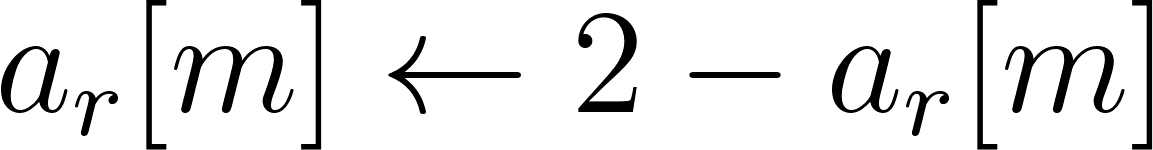](https://www.codecogs.com/eqnedit.php?latex=a_r%5Bm%5D%5Cleftarrow%202-a_r%5Bm%5D#0)  Then center (around 0): [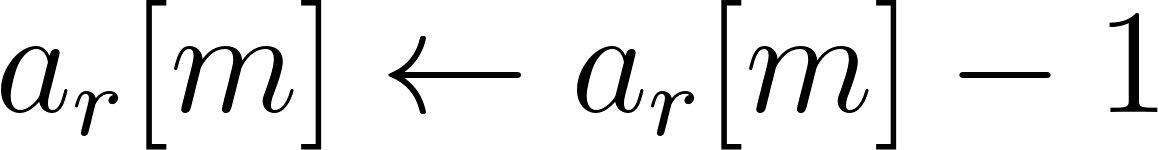](https://www.codecogs.com/eqnedit.php?latex=a_r%5Bm%5D%5Cleftarrow%20a_r%5Bm%5D-1#0)  Amplify: [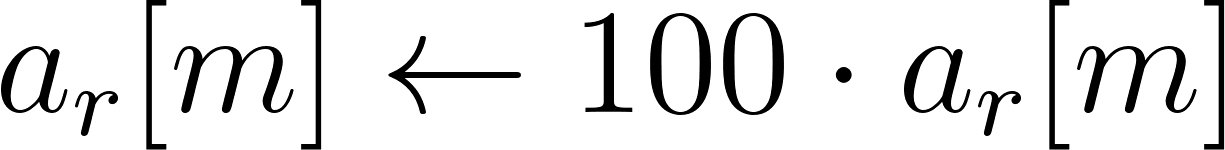](https://www.codecogs.com/eqnedit.php?latex=a_r%5Bm%5D%5Cleftarrow%20100%20%5Ccdot%20a_r%5Bm%5D#0) |
| Step-5 | : | Threshold [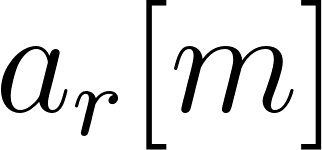](https://www.codecogs.com/eqnedit.php?latex=a_r%5Bm%5D#0):  Let [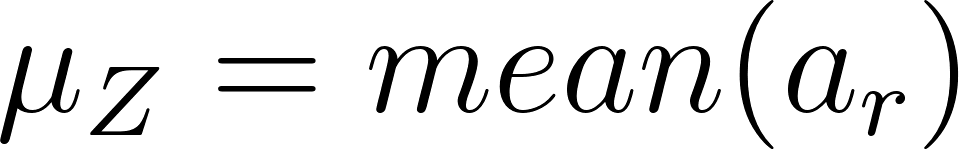](https://www.codecogs.com/eqnedit.php?latex=%5Cmu_Z%3Dmean(a_r)#0), [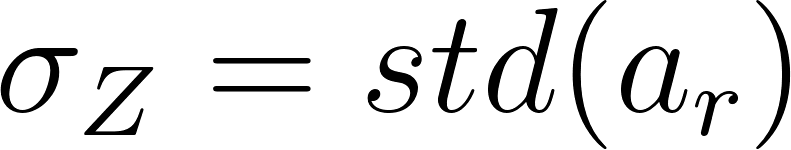](https://www.codecogs.com/eqnedit.php?latex=%5Csigma_Z%3Dstd(a_r)#0)  Define threshold: [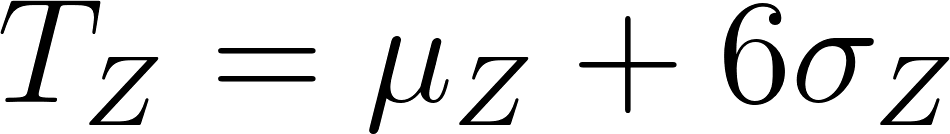](https://www.codecogs.com/eqnedit.php?latex=T_Z%20%3D%20%5Cmu_Z%2B6%20%5Csigma_Z#0)  Set [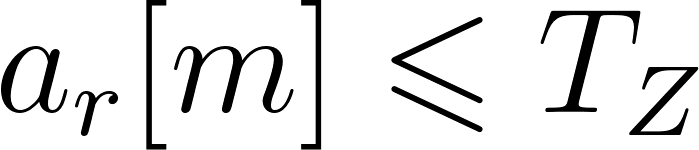](https://www.codecogs.com/eqnedit.php?latex=a_r%5Bm%5D%5Cleqslant%20T_Z#0)  Also clip: [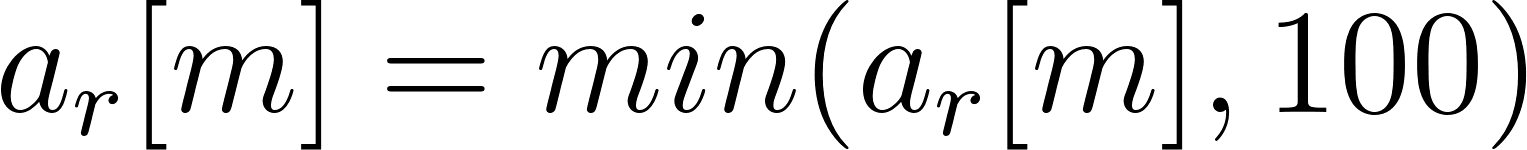](https://www.codecogs.com/eqnedit.php?latex=a_r%5Bm%5D%20%3D%20min(a_r%5Bm%5D%2C100)#0) |
| Step-6 | : | Similarly, process [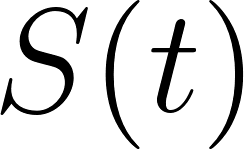](https://www.codecogs.com/eqnedit.php?latex=S(t)#0):  Scale: [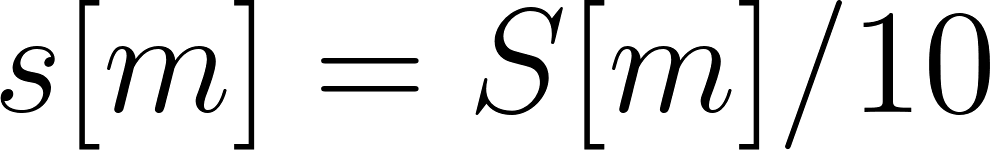](https://www.codecogs.com/eqnedit.php?latex=s%5Bm%5D%20%3D%20S%5Bm%5D%2F10#0)  Compute threshold: [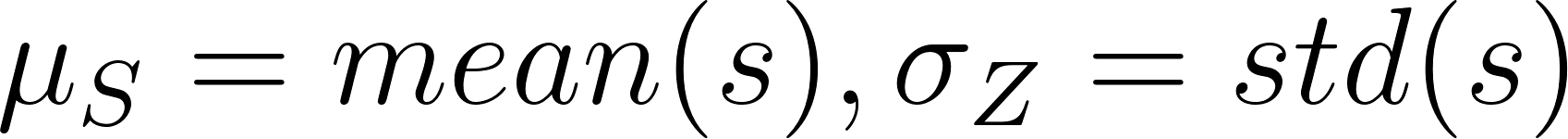](https://www.codecogs.com/eqnedit.php?latex=%5Cmu_S%3Dmean(s)%2C%20%5Csigma_Z%3Dstd(s)#0)  Set [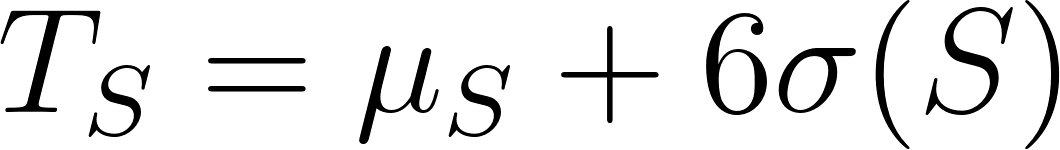](https://www.codecogs.com/eqnedit.php?latex=T_S%3D%5Cmu_S%2B6%20%5Csigma(S)#0)  If [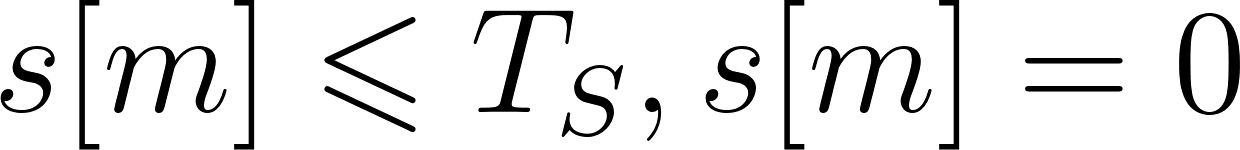](https://www.codecogs.com/eqnedit.php?latex=s%5Bm%5D%5Cleqslant%20T_S%2C%20s%5Bm%5D%3D0#0)  Clip: [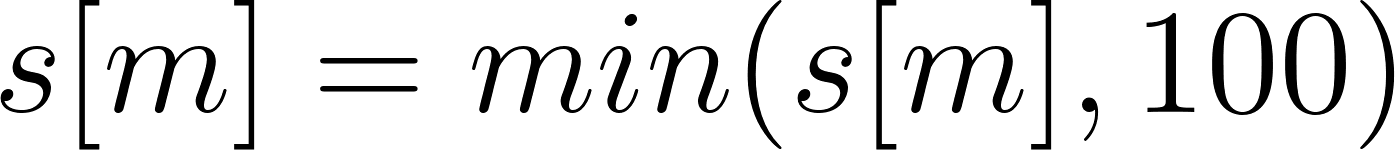](https://www.codecogs.com/eqnedit.php?latex=s%5Bm%5D%20%3D%20min(s%5Bm%5D%2C100)#0) |
| Step-7 | : | Apply Hann window to both signals: [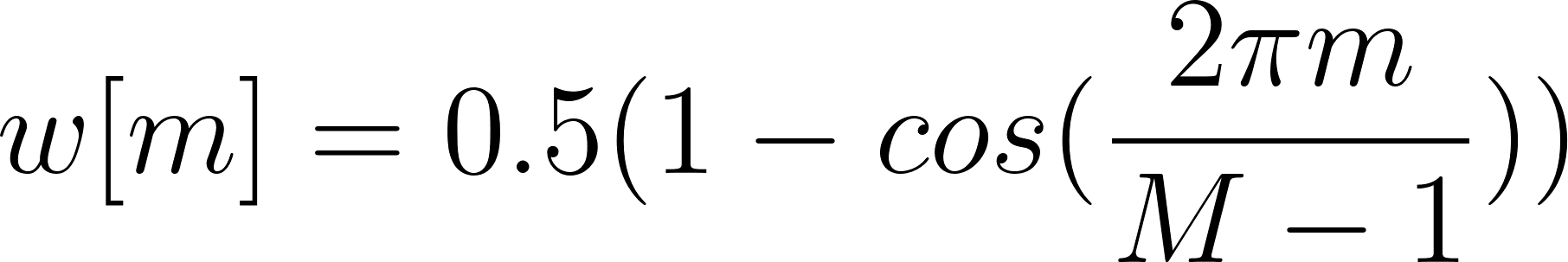](https://www.codecogs.com/eqnedit.php?latex=w%5Bm%5D%3D0.5(1-cos(%5Cfrac%7B2%5Cpi%20m%7D%7BM-1%7D))#0)  Windowed signals: [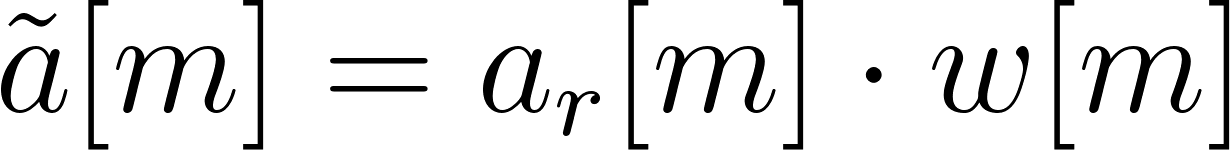](https://www.codecogs.com/eqnedit.php?latex=%5Ctilde%7Ba%7D%5Bm%5D%3Da_r%5Bm%5D%5Ccdot%20w%5Bm%5D#0), [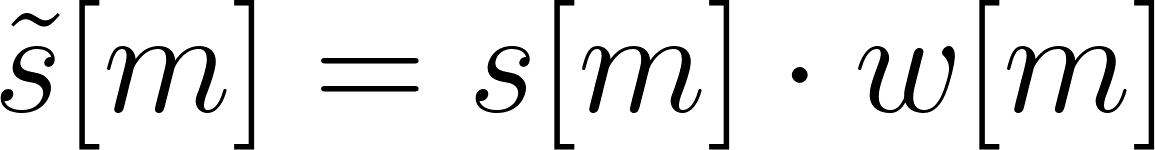](https://www.codecogs.com/eqnedit.php?latex=%5Ctilde%7Bs%7D%5Bm%5D%3Ds%5Bm%5D%5Ccdot%20w%5Bm%5D#0) |
| Step-8 | : | Compute cross-correlation between [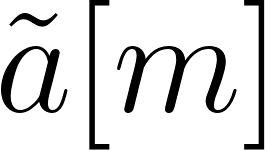](https://www.codecogs.com/eqnedit.php?latex=%5Ctilde%7Ba%7D%5Bm%5D#0) and [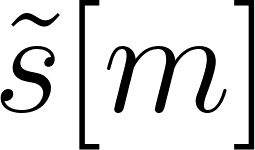](https://www.codecogs.com/eqnedit.php?latex=%5Ctilde%7Bs%7D%5Bm%5D#0) with positive lags only:  Let [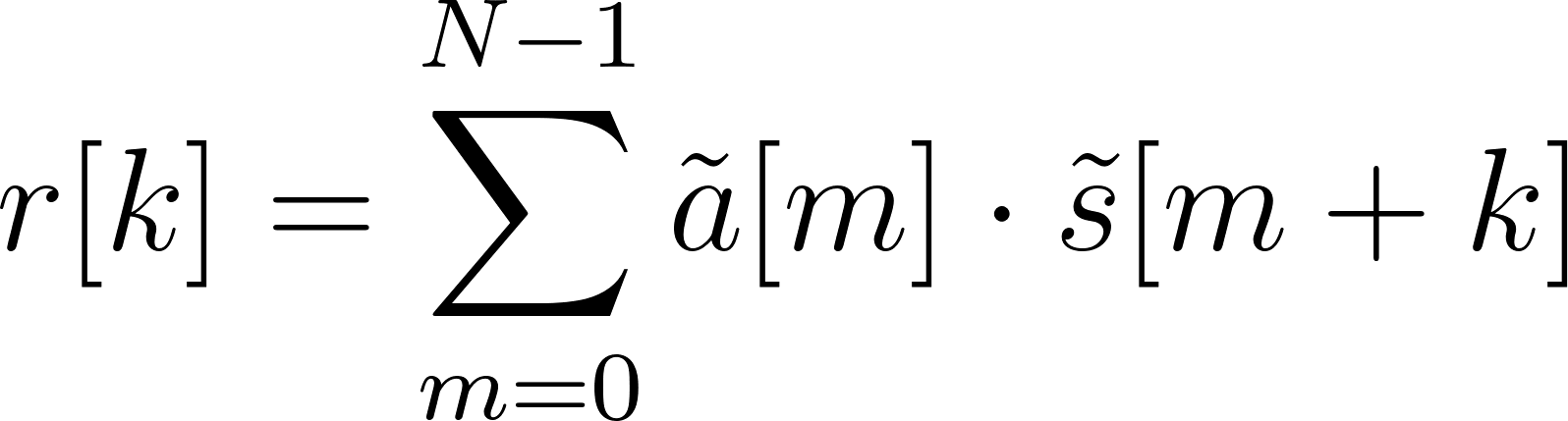](https://www.codecogs.com/eqnedit.php?latex=r%5Bk%5D%3D%5Csum_%7Bm%3D0%7D%5E%7BN-1%7D%5Ctilde%7Ba%7D%5Bm%5D%20%5Ccdot%20%5Ctilde%7Bs%7D%5Bm%2Bk%5D#0) for [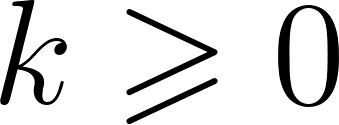](https://www.codecogs.com/eqnedit.php?latex=k%5Cgeqslant%200#0) |
| Step-9 | : | Identify lag with maximum correlation: [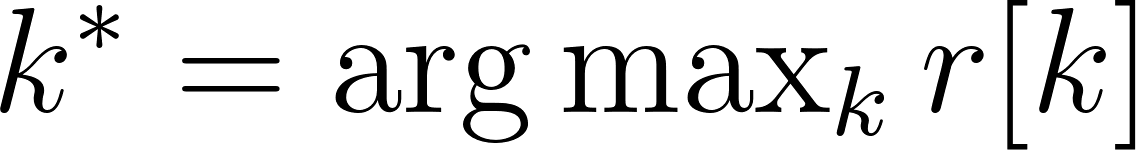](https://www.codecogs.com/eqnedit.php?latex=k%5E*%3D%5Carg%7B%5Cmax%7D_%7Bk%7D%5C%2Cr%5Bk%5D#0) |
| Step-10 | : | Convert lag to time: [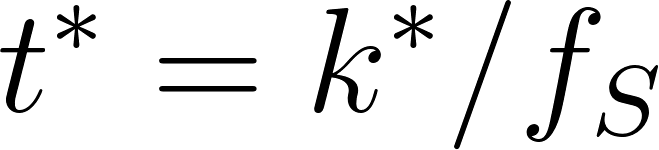](https://www.codecogs.com/eqnedit.php?latex=t%5E*%3Dk%5E*%2Ff_S#0) |
| Step-11 | : | Return [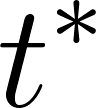](https://www.codecogs.com/eqnedit.php?latex=t%5E*#0) |

Please note that Algorithm 2.1 assumes that both [
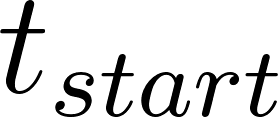
](https://www.codecogs.com/eqnedit.php?latex=t_%7Bstart%7D#0) and [
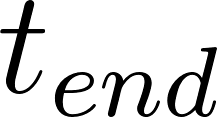
](https://www.codecogs.com/eqnedit.php?latex=t_%7Bend%7D#0) moments are present in [
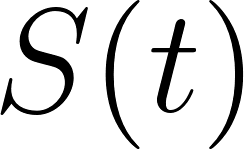
](https://www.codecogs.com/eqnedit.php?latex=S(t)#0) and[
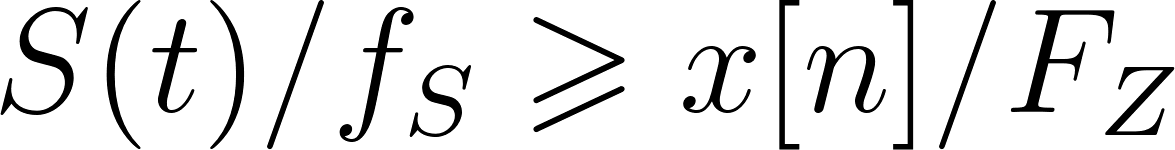
](https://www.codecogs.com/eqnedit.php?latex=S(t)%2Ff_S%20%5Cgeqslant%20x%5Bn%5D%2FF_Z#0). Data that does not satisfy these conditions cannot be aligned using this method. Upon applying it to 64 nights of Zmax and Somnoscreen data from the Wearanize+ dataset, we found that [
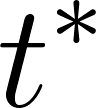
](https://www.codecogs.com/eqnedit.php?latex=t%5E*#0) identified by the algorithm was within the same (30-second) epoch as the manually identified Lights Out moment for 58 nights (90.63%) and within the next epoch (one epoch lag) for 62 nights (96.88%). The algorithm relies on identifying matching peaks in the signals; hence, the absence of such features is the primary factor behind its failure to accurately identify [
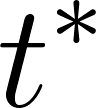
](https://www.codecogs.com/eqnedit.php?latex=t%5E*#0). Furthermore, in the Wearanize+ project, participants turned both devices on right before going to bed and turned them off soon after waking up, meaning that neither device captured extensive waking activity—typically reflected as irregular bursts of high-amplitude peaks in accelerometer signals. Aligning signals with substantial waking activities may be challenging for such threshold-based methods.

## Appendix 3. Wearanize+_raw_v1.0 Organization

Here, we present a brief description of the organization of the information provided in the Wearanize+ raw dataset described in Section 3.1. These are the directories within the file *Wearanize+_raw_v1.0.zip*:

- ***1.Raw_data***: contains the raw data collected by the devices in an unaltered form. However, to ensure compatibility and ease of use, data has been provided in cross-platform formats (EDF and CSV). In the interest of saving space and keeping the dataset compact, redundant files (without any useful data) have been deleted.
- ***2.Sleep_scores*:** contains multiple sets of sleep scores, each segmented into 30-second epochs and labeled from −1 to 4, representing Artifact, Wake, N1, N2, N3, and REM, respectively. The data is organized in the following sub-directories:
  - ***1.PSG_manual_scores***: contains sleep stages manually identified by an experienced scorer who reviewed the PSG data. Each subject’s scores have been provided in an individual TXT file featuring two columns: the first one with the identified sleep stages (0–4, with −1 indicating unscorable epochs) and the second one indicating arousals (0 for none, and 1 for present). This set of sleep scores would be considered the ground truth for most studies.
  - ***2.PSG_autoscores_U-Sleep_v2.0***: contains autoscores generated by the widely used U-Sleep v2.0 model, provided in subject-specific CSV files. Each file features two columns: the identified sleep stages and their associated probabilities, expressed in %.
- ***3.Manual_synchronization***: contains an Excel sheet detailing the synchronization points for each device *Zmax_start_sec* and *Zmax_end_sec* denote the Lights Out and Lights On moments (in seconds), which were automatically identified by eegFloss and used as boundary points for synchronization. *PSG_start_sec*, *PSG_end_sec*, *Emp_start_sec*, *Emp_end_sec*, *Actpal_start_sec*, and *Actpal_end_sec* represent the identified boundary points for the corresponding subject’s PSG, Empatica, and Activpal recordings, respectively. A negative value in any column that represents starting points suggests an offset, indicating that the Lights Out moment was not captured by the corresponding device. In such instances, the recording should be padded at the beginning to align with the duration of the Zmax recording. Conversely, a positive value in *PSG_offset* suggests that the Lights On was not captured by the PSG device, requiring padding at the end to match Zmax’s duration. The count of seconds begins at 1, meaning the first data point is recorded at (1+1/sampling_rate) seconds. A “0” in both the starting and ending points indicates that the recording was unavailable, while a “-999” denotes that the recordings could not be synchronized with Zmax due to a lack of match in observed events.
- ***4.Demographic_info_and_questionnaires***: contains the participants’ demographic information and their responses to the three standard questionnaires (PSQI, MADRE, and PHQ-9) in an Excel file. Please see the *field options* and *study variables* sheets for details.
